# Carbon Capture and Utilization for Sustainable Supply Chain Design of Intermediate Chemicals: The Formate Factory

**DOI:** 10.1021/acssusresmgt.4c00472

**Published:** 2025-03-07

**Authors:** Ariane Silveira Sbrice Pinto, Nalan Gulpinar, Fang Liu, Elizabeth Gibson, Linsey Fuller, Philip Souter

**Affiliations:** † Business School, Management Department, 3057 Durham University, DH1 3LB Durham, England, United Kingdom; ‡ School of Natural and Environmental Science, Newcastle University, NE1 7RU Newcastle-upon-Tyne, England, United Kingdom; § 8552 Procter and Gamble, Newcastle Innovation Centre, Whitley Road, Longbenton, Newcastle upon Tyne NE12 9TS, England, United Kingdom

**Keywords:** carbon capture and utilization; life cycle assessment, techno-economic analysis, formate production, biocatalysis, electrocatalysis, supply chain design

## Abstract

Carbon capture and use technologies (CCUt) to valorize industrial flue gases into products is the key to a circular economy. Risks related to technology readiness level (TRL) and supply chain design challenges still lack clarity, however, which might hinder the widespread implementation of CCUt. Industrial decarbonization requires a holistic approach, that includes supply chain design, techno-economic analysis (TEA), and lifecycle assessment (LCA) to drive the transition toward a low-carbon future. Here, formate production with biocatalytic (BR) or electrocatalytic (ER) routes was evaluated as a potential CCU strategy for industrial decarbonization. Electrocatalysis typically had a lower production cost than biocatalysis manufacturing, while the product carbon footprint (PCF) was generally lower for biotechnology. The uncertainty analysis (UA) indicated 58% and 2% probability to reduce emissions below petrochemical emissions with the BR and ER, respectively. Strategies for facilitating the deployment of formate factories, including carbon trading schemes, creating a market for industrial flue gas, and/or producing lower-grade products, were discussed.

## Graphical abstract

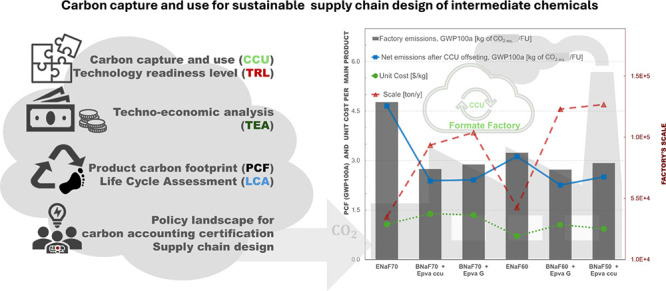

## 1 Introduction

Carbon dioxide serves as a valuable feedstock for the production of intermediate chemicals. The mitigation of global warming occurs by reducing process’ emissions and the reliance on petrochemical feedstocks.^1–3^ Embracing sustainable supply management by converting CO_2_ into chemicals can be particularly advantageous for sustainable development since a high quantity of CO_2_ from steelmaking flue gases can be used as feedstock, potentially mitigating up to 2.4% of total greenhouse gas emissions in the United Kingdom (UK) only.^4^ In particular, the design of the formate supply chain is crucial for decarbonizing fast-moving consumer goods. This intermediate chemical has a significant market value (550 million USD by 2032)^5^ with a wide range of applications, including food additives, de-icing agents, liquid (organic) hydrogen carrier, and enzyme stabilizers in detergents.^6,7^ Biochemical^8^ and electrochemical^9^ technologies have emerged as leading CCUt. However, further strategies for large scale production must be explored since the economic feasibility has been often hindered by low yields, high auxiliary material consumption, and significant energy demand during the manufacturing of both BR and ER technologies.^10,11^

In BR, the synergy among formate dehydrogenases (FDHs) and hydrogenlyases (FHL)^8^ to catalyze the interconversion of H_2_, CO_2_, and formate has been suggested^12,13^ to overcome byproducts production from fermentation processes.^14^ High specificity of enzymes combined with encapsulation leads to low replenishment costs, reuse over different reaction cycles, easy separation, and increase of stability over time of biocatalysts^15^ in a continuous process.^16,17^ Immobilized enzymes combined with BFG from steelmaking as feedstock indicated the potential of a large scale operation under atmospheric conditions, resulting in 18 mM formate (pH = 6.5, T = 30 °C, 48 h).^6,8^ Mass transfer resistance was mitigated by operating with high-pressure systems (P > 2 bar), increasing substantially the yields up to 500 mM^14,18^ as a result of a high concentration of gaseous feedstock in the liquid broth. Besides, the production of enzymes can use solely formic acid-CO_2_ ^19,20^ as a carbon source instead of glucose^21^—an opportunity for launching a CCU factory with 100% of carbon from CO_2_. Despite its potential attractiveness for industrial-scale applications, the technical design of using immobilized enzymes to convert flue gases into formate still have low TRL (2–3), and further insights on techno-economic-environmental aspects of a large-scale production shall be beneficial to guide research and development (R&D) investments.

On the other hand, significant progress has been made in designing ER. Typical design varies with the reactor type-catalyst,^9^ where electrolytes, faradaic efficiency (50 to 100%), current density (0.4–1.0 A cm^–2^), alkaline/neutral pH, and cell voltage (0.6 to 5.0 V)^22^ have been optimized. The yield of formate can lead to 1.2 M (50 h) product at the cathode chamber with aqueous solution of (bi)­carbonate.^23^ Moreover, the pilot-/semi-pilot scalability of the electrochemical technology has been further developed^24^ and tested for up to 40,000 cm^2^ stacks and high stability with current densities of 1.0 A cm^–2^.^23,24^ Outstanding savings in the product carbon footprint (PCF_CCU_ < 80%)^25^ also show opportunity for decarbonizing the formate supply chain. The electroreduction of CO_2_ offers higher TRL (>4) than the biochemical pathway since pilot scale tests converted 146 kg of CO_2_/day into 110 kg of formate/formic acid/day,^22^ while the biocatalytic reactor capacity produced less than 1 kg/day of formate from industrial flue gases.^8^

The economic outlook for converting CO_2_ into chemicals^10,11^ indicates that even optimal technical assumptions would still require low energy costs (<0.07 USD/kWh) and low CO_2_ cost (commodity price <40 USD/MT) for achieving profitability in both electrocatalysis and fermentation CCU approaches. However, in the context of a stakeholder’s decision making, a comparison of the technologies is not straightforward since modelling assumptions for forecasting large scale material/energy flows^26^ and other economic-environmental assumptions^27,28^ can limit the comparison of different literature. Furthermore, a discussion around technology innovation and United Nations sustainability goals (SDGs)^2^ are still not clear for the formate manufacturing with ER and BR.

Here, techno-economic-environmental aspects of producing formate with electro-/bio-catalysis were compared, identifying key opportunities and bottlenecks for the UK landscape. Policy interventions and investment considerations were discussed transparently to support sustainable resource management. Future insights included a prospective analysis, aligning CCU supply chain design with Paris Agreement targets.^29^ Uncertainties of unit cost and the PCF were forecasted with kernel density estimation (kde).

## 2 Results and Discussion

### 2.1 Techno-Economic Analysis

The formate factory proved to be economically viable for CCUt under certain circumstances. Net Present Value (NPV), revenue, total capital of investment (TCI), capital (CapEx), and operational expenditures (OpEx) of producing high-/low-grade sodium (NaF) and potassium formate (KF) (*i* ^th^ mass grade (ENaF_*i*_ and EKF_*i*_, respectively) are summarized in Figure 1.

**Figure 1:**
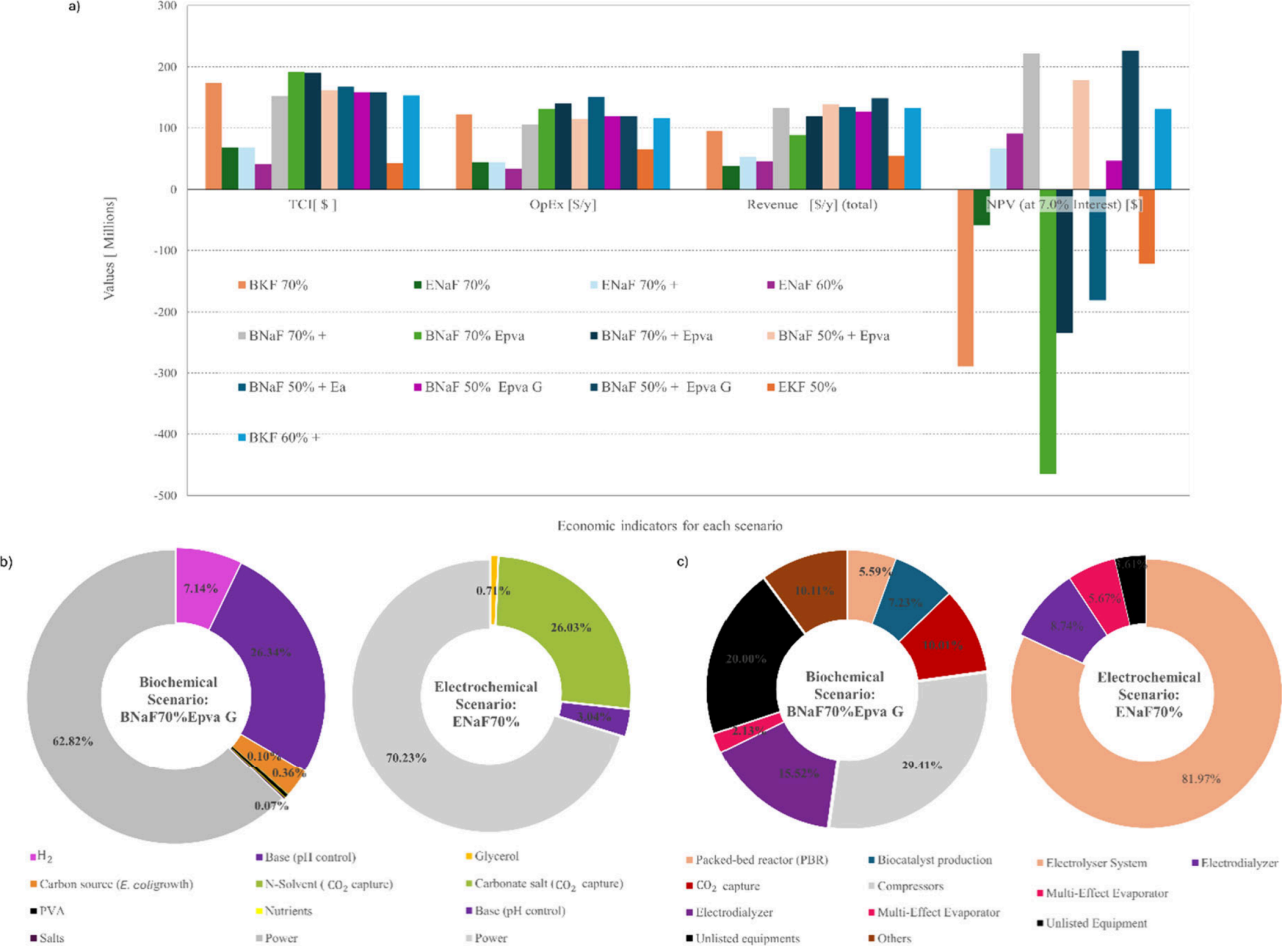
Techno-economic analysis. NPV, TCI, revenue, OpEx, and CapEx (a). Breakdown of OpEx (b) and CapEx (c). Additional income from BFG treatment was indicated by the + sign. E_PVA_ and *E*_a_ indicated immobilization in PVA and alginate supports, respectively. Glucose (G) and formate-CO_2_ (CCU) were used as the carbon source for bacteria growth.

Feasible production of high-grade formate (70% wt), in both the ER (ENaF 70%+) and BR (BNaF 70%+, BKF60%+) manufacturing strategies (Figure 1-a), was achieved if additional revenue from BFG treatment is considered as complementary income. To achieve profitability without applying this strategy and operating with yields associated with the current TRL, lower-grade products could be an alternative, as deemed in ENaF60% and BNaF50% E_PVA_-_G_. To analyze the trade-off between expenses and income, the breakdown of OpEx and CapEx was displayed in Figure 1-a and -b, respectively.

For both CCUt, utilities and raw materials accounted for significant portions of OpEx (Figure 1-b), totaling, on average, 20% and 40% of the total, respectively. A closer examination of raw materials expenses revealed that power demand (utilities, electricity) was the main cost driver, reaching up to 70% and 63% of OpEx for ER and BR, respectively. This result was expected since low-grade products indicated potential for economic feasibility (Figure 1-a).

In fact, the BR required additional auxiliary materials: organic solvents, polymers, H_2_, and nutrients for bacterial growth. Extra costs associated with enzyme replenishment, for instance, led to unprofitable scenarios if an alginate bed was considered, even for low-grade formate solutions (50% wt), as indicated by BNaF 50%^+^ *E*_a_. Besides, the use of BFG in the BR required absorbers and stripping columns to provide CO_2_-rich feed to biocatalytic reactors (PBRs)—which mitigates the deactivation of biocatalysts by other gases.^30^ Moreover, the consumption of an alkaline solution was significant exclusively for BR. In contrast, the ER only uses electrolytes as auxiliary materials, and the absorption system was not necessary. Particularly, carbonate salts were a notable expense, representing 26% of the OpEx.

Other OpEx includes facility dependent costs, which are mainly driven by CapEx.^31^ The CapEx of equipment purchase was the highest contributor to the TCI for both technologies, totaling almost 30% (Figure 1-c). For BR, PBRs, the CO_2_ capture system (compressors, absorber, and stripping column), electrodialyzer, and biocatalyst production contributed 6%, 39%, 16%, and 7% in CapEx, respectively. The electrolyzer, electrodialyzer, and multi-effect evaporator had the highest contributions to the CapEx of the ER, reaching 82% and 9%, respectively. The CapEx for reactors and the catalyst production in the BR system had a lower impact on TCI than the electrolyzer facility in ER factory.

### 2.2 Life Cycle Assessment

LCA of formate production was conducted across nine impact categories, which indicated better overall performance for BR than ER- as displayed in Figure 2. The comparison of burdens and benefits of different production systems showed that low-grade products (ENaF50, BNaF50_ccu_, BNaF60_G_) had lower environmental impacts than high-grade ones for global warming (GWP100a/PCF), freshwater ecotoxicity (FTP), freshwater eutrophication (FEP), marine eutrophication (MEP), terrestrial eutrophication, abiotic depletion (ADP), water use (WUP), and acidification (ACP) potentials, except for the ozone depletion potential (ODP).

**Figure 2:**
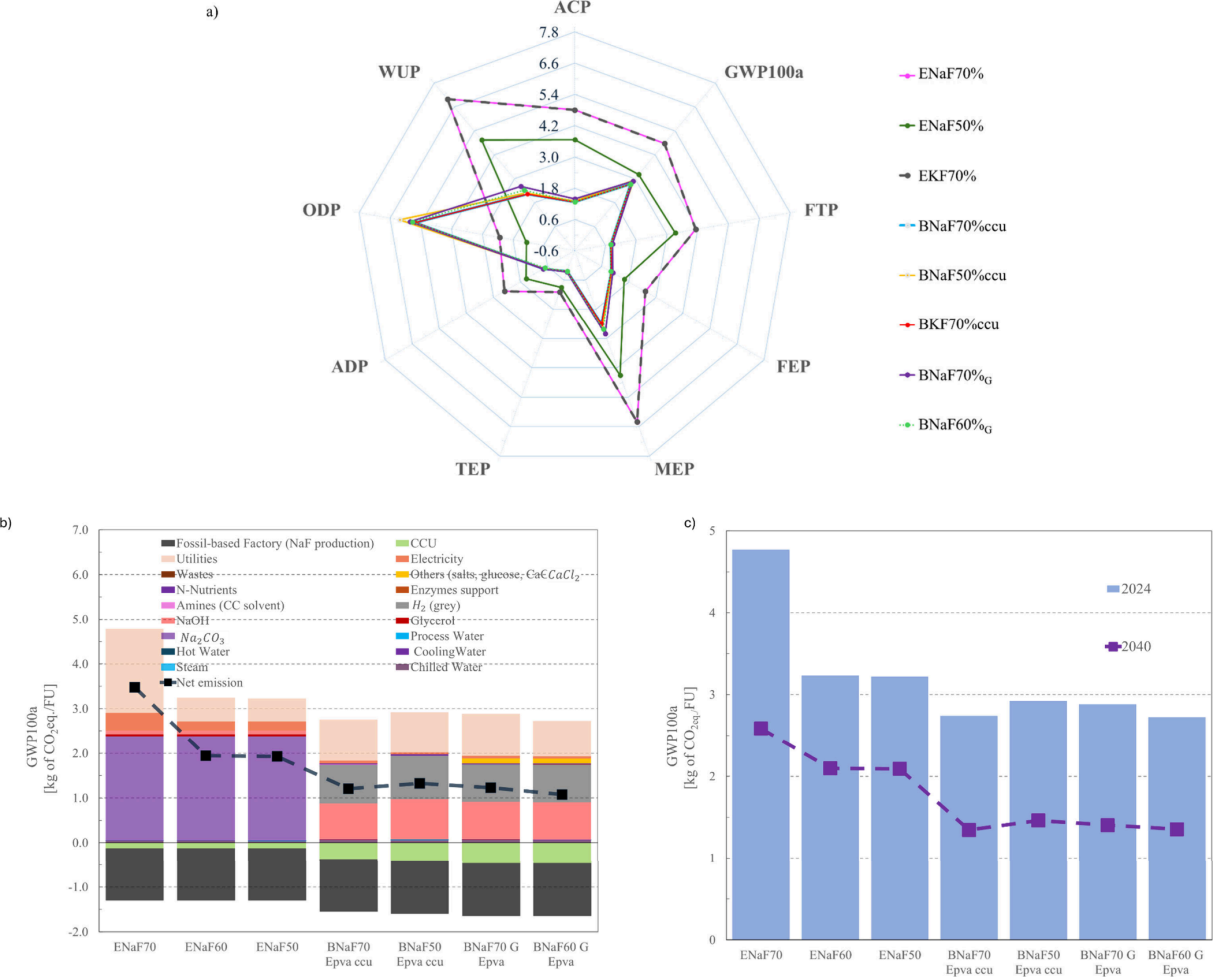
Environmental analysis. LCA (a). Breakdown of the PCF considering emissions without and with offsetting (b). Prospective LCA for 2040 including emissions without a CCU offset for 2024 (reference year) and 2040 (c). Net emissions with carbon offsetting (PCF_Net,_ net emissions line) indicated the impact on total GWP100a after avoiding the direct release of BFG into the atmosphere and mitigating the consumption of resources from the petrochemical production of formate (b).

To hotspot bottlenecks and opportunities for supply chain design, the breakdown of the PCF (Figure 2-b) was analyzed.

To account for the benefit of locking the carbon in the final product and avoiding atmosphere emissions of the BFG, the carbon offsetting was analyzed. PCF_Net_ showed a carbon offsetting of 77% and 33% for ER and BR, respectively. Both technologies showed a significant power demand for utilities. However, the electricity requirement was more pronounced in ER compared to BR. Indeed, high energy demands in the ER process have been the key challenge when converting CO_2_ into formate or formic acid^32^—particularly for the ENaF70% scenario.

The impact of raw materials on the PCF varied by technology, except for the alkaline solution, which remained consistent. The replenishment of carbonate salts had one of the highest impacts on the PCF in the ER process, while H_2_ was particularly important in the BR process. Although gray-H_2_ was used as the supply, lower emissions are expected with the development of electrolytic and/or low-carbon technologies in a 10 to 20 year timeframe.^33,34^ Surely, the design of the supply chain will be essential to mitigate industrial emissions, not just by replacing H_2_ technologies but also by including green strategies for manufacturing intermediate chemicals.

To meet the targets set by the Paris Agreement,^29^ significant socioeconomic changes in current industrial processes must occur. Examples of decarbonization strategies include improving the efficiency of low-carbon technologies and transitioning from fossil-based to renewable energy sources. To forecast the impact of reducing the embodied carbon of background processes, historical trends were extrapolated using the PREMISE^35^ framework. Projections of the PCF for the 2040 timeframe are shown in Figure 2-c. If the increase in global mean surface temperature by 2,100 remains below 1.6–1.8 °C of pre-industrial levels, the comparison of 2024 and 2040 landscapes indicated potential benefits on producing formate with CCUt. The mitigation of GWP100a can be up to half 2024’s emissions by 2040 (Figure 2-c).

### 2.3 Uncertainty

#### 2.3.1 Economics

The location for launching CCU factories should be also chosen with caution, considering costs/availability of feedstock, energy, and raw materials. Energy costs, for instance, were a significant cost for both technologies. The comparison of different regions, using the price of UK’s energy supply as reference, indicated that the highest savings in OpEx would be expected in China (<77%), followed by Brazil (<60%), the USA (49%), and the EU (43%).^36^ In EU countries, for instance, the range of energy cost can vary from 0.20 to 0.5 EUR/kWh,^37^ whereas in the USA^38^ and China,^46^ power costs for industrial processes can be reduced, being estimated between 0.07 to 0.10 USD/kWh from 2020 to 2023. Although the low price of energy in developing countries, such as Brazil, can be promising, further investments in CCU policy might be necessary, mainly in infrastructure for designing the supply chain. From an energy perspective, the USA and China may provide better opportunities for CCU sites than the EU region.

The combination of uncertainties on supply/demand of resources combined with cost variation might impact the unit production cost, as displayed in Figure 3.

**Figure 3:**
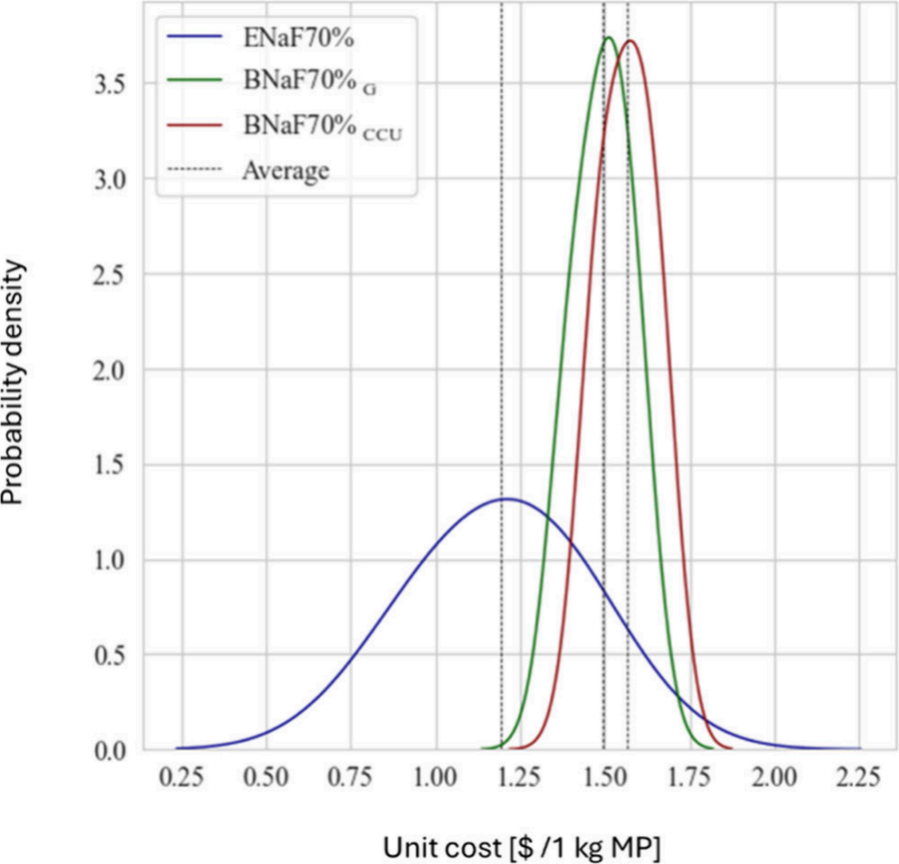
Unit cost uncertainty.

The choice of different technologies directly affects the final yields and demand for energy and auxiliary materials. As a result, the unit cost of the final products varies across scenarios. The average selling price was estimated in 1.25, 1.50, and 1.60 for ENaF70%, BNaF70%G, and BNaF70%_CCU_, respectively. Here, the minimum average production cost was projected in, approximately, $1.25/kg of MP. Recently, formate’s selling price for CCUt was forecasted in $0.96/kg MP^32^—which corroborates with the Figure 3 outcome. A detailed discussion about raw materials costs is included in the Supplementary Information (SI).

#### 2.3.2 Product Carbon Footprint

The probability of reducing emissions below the petrochemical baseline with CCUt was 58% and 2% for the BR and ER, respectively, as indicated in the hachured area of Figure 4-a. The sensitivity analysis (Figure 4-b, c, and d) indicated the power consumption (utilities and electricity) as the highest contributor for emissions, leading to over 90% of the PCF’s uncertainty. Electrolytes and H_2_ consumption were also relevant for uncertainties.

**Figure 4:**
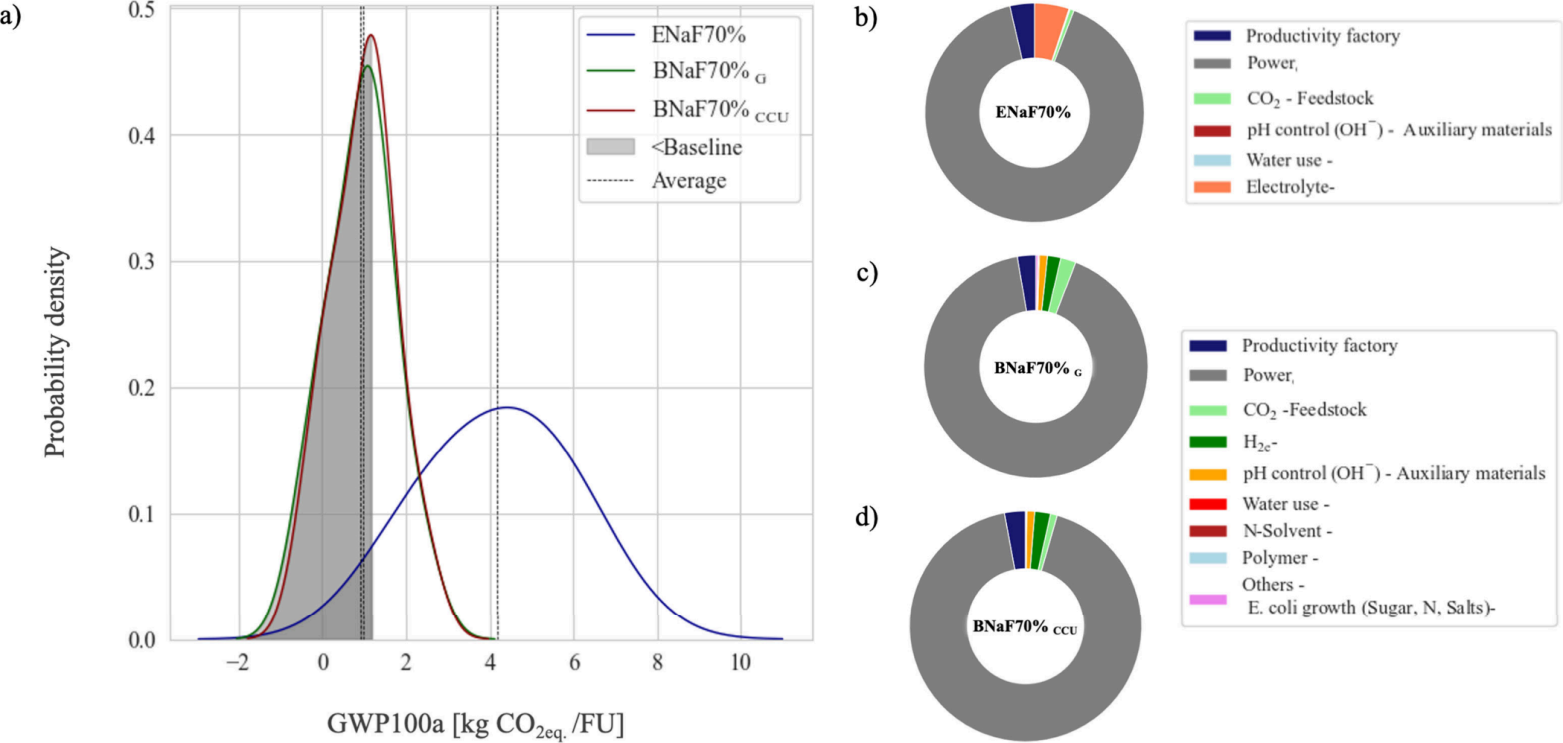
PCF’s uncertainty. Comparison of the probability density for formate production (grade 70% wt) for different technologies (a). First order Sobol index for (b) ENaF70% and BNaF70% (c) G and (d) CCU.

The resolution was constrained by the TRLs of both BR (2-3) and ER (>4) technologies. Process optimization and control could shift these thresholds in either direction. For achieving an equivalent TRL for both technologies (>4), further R&D for BR shall focus particularly in PBR design and biocatalyst production.^39^

In fact, scaling up challenges will impact both unit cost and the PCF, emphasizing the need for further research to ensure practical cost scalability for deploying a green market for formate with CCUt.

### 2.4 Policy for Supply Management

Future supply chain design shall focus on SDGs,^2^ as partnerships; innovation, resource efficiency/management (clean and affordable energy/feedstocks); infrastructure; market design; and regulation.

#### 2.4.1 Stakeholders’ Engagement: Partnerships for Sustainable Development

Government engagement with industrial clusters can accelerate the circular economy and support net-zero targets.^40^ In the UK, the government aims to capture 20–30 MtCO_2_/year by 2030 with £26 million already invested in CCUS innovation.^41^ Beyond government support, private sector collaboration will be essential for scaling up CCU technologies. Industries such as steelmaking, energy, food additives, de-icing, and detergents should collaborate to establish a green market for formate.

#### 2.4.2 Innovation

Innovation can reduce formate’s unit cost and the PCF. The analysis of the large-scale production highlighted opportunities for investments in product quality and process development.

##### Product Quality

As a precursor to a variety of other products, formate could be available in lower grades for specific sectors. Trade-offs between final product grade and profitability indicated significant reduction in energy use during the purification process, which reduced both OpEx and the PCF.

##### Process Development

Due to low TRL, further investments in research and development would be beneficial for both ER and BR. Improving productivity/yield,^8,42^ enhancing energy efficiency,^22^ and increasing (bio^15,43^ ) catalyst^22,24^ stability are still necessary. In general, for both technologies, integrating the processes in pilot scale would be beneficial for validation of the manufacturing process and data gathering to optimize and forecast the impact of scaling up on yields, losses, and other parameters. An outline for research opportunities was summarized in Table 1.

**Table 1:** Research Opportunities

Biocatalytic route	Electrocatalytic route
Design reactors under pressurized conditions	Enhance stability of the catalysts over time
	Study maximum hours/cycles of use
Investigation of low-cost and eco-friendly polymers to enhance stability/activity of the enzymes	Investigation of control strategies to mitigate salt precipitation
Enhance yields of both biomass and enzymes from *Escherichia coli* during its growth or forecast other bacteria to produce enzymes with CO_2_ -formate as the carbon source	Data gathering to forecast potential costs of catalyst replenishment/disposal per amount of product

#### 2.4.3 Infrastructure and Resource Management

The first stage of supply chain development involves the establishment of infrastructure to operate with CO_2_ as feedstock, which can be captured directly from the emission source or be provided from future geological storage sites. Policymakers and stakeholders should also consider if reducing the overall flue gas emissions could affect the longevity of CCU industrial sites due to insufficiency of feedstock supply. The utilization of geological sources or distribution in pipelines must consider regulation for CO_2_-grade. In the context of producing intermediate chemicals, diluted concentrations or the presence of impurities could hamper economic feasibility of CCU factories. In the UK, non-pipeline transport^44^ projects have been proposed, and policies have been evaluated^45^ in collaboration with companies, trade bodies, scientists, and private investors.^46^

Besides, carbon neutrality and economic feasibility with CCUt might require additional strategies. The formate production, for instance, was strongly linked to energy and H_2_ supplies. In alignment with SDGs,^2^ investments in green and affordable energy supply were demonstrated to be crucial for both ER and BR. Integrating CCU factories to existing industrial sites would also mitigate the energy demand after integration heat exchanges. Designing the H_2_ supply chain to produce this commodity with low embodied-carbon and affordable market prices will also be also essential. In the UK, contracts have been awarded^41^ to move forward to commercial deployment of H_2_ manufacturing.

#### 2.4.4 Market Landscape

Financial market regulation, emissions trading systems (ETS), foreign affairs,^45^ and industrial development will be critical for CCUt deployment.^47^ Transforming industrial flue gases into chemicals can be framed as waste treatment, generating revenue to ignite investments in CCUt, similarly to the scenarios considered for formate production, where a market was created for industrial flue gases. Alternatively, the capacity of capturing/avoiding CO_2_ emissions of a CCU factory could also be used in ETS to bring economic benefits.

Successfully applied in power and energy-intensive sectors in the EU and USA,^48,49^ the goal of ETS is to meet the Kyoto target^49^ by tracking emissions, applying penalties of non-compliance of the decarbonization plan, and trading allowances. In a CCU factory, green certification could monitor emissions for trading allowances, igniting the deployment of CCU factories by reducing economic risks. The international sustainability and carbon certification (ISCC)^50^ accounts for carbon savings by comparing the net emissions of the CCUt to a benchmark/baseline. Likewise, this approach has been rigorously applied for biofuels.^51^

#### 2.4.5 Regulation

Regulation with the LCA can be particularly challenging at early TRLs, making clear regulatory objectives essential. Policymakers must balance environmental integrity, cost-effectiveness, and local suitability while addressing the complexities of LCA in a circular economy context.^27,52^ The flexibility of LCA modelling can result in negative or carbon neural emissions depending on the scope because it defines material/energy flows, cut offs, and allocation of the benefits burdens.^27,28^ Then, a standardized modelling and fair allocation of burdens/benefits will be critical^27^ to prevent double counting of benefits along the supply chain for either biogenic or post-combustion CO_2_ utilization. Alongside, relying solely on the PCF may not capture the full environmental impact of new manufacturing processes since trend disparities among the impact categories might occur, as observed for the case of the ODP in formate’s LCA. Although regulation for sustainable development shall consider environmental, social, and governance extents over time, social and temporal aspects were out of the scope of this work.

In short, accountability, transparency, robustness, and compatibility across jurisdictions and industries^27,28^ shall be prioritized to avoid greenwashing, double counting, and carbon leakage.^45^

## 3 Methods

### 3.1 Scope and System Boundaries

A techno-economic-environmental assessment of biochemical and electrochemical formate production from BFG was conducted using an *ex-ante* approach with experimental data from previous studies.^15,6,8,19,20,23^ NPV evaluated economic performance, while LCA burdens and benefits were assessed (2024, 2040). Cradle-to-gate system boundary was applied. The functional unit was with 1 kg MP. The block diagram for each technology was presented in Figure 5.

**Figure 5:**
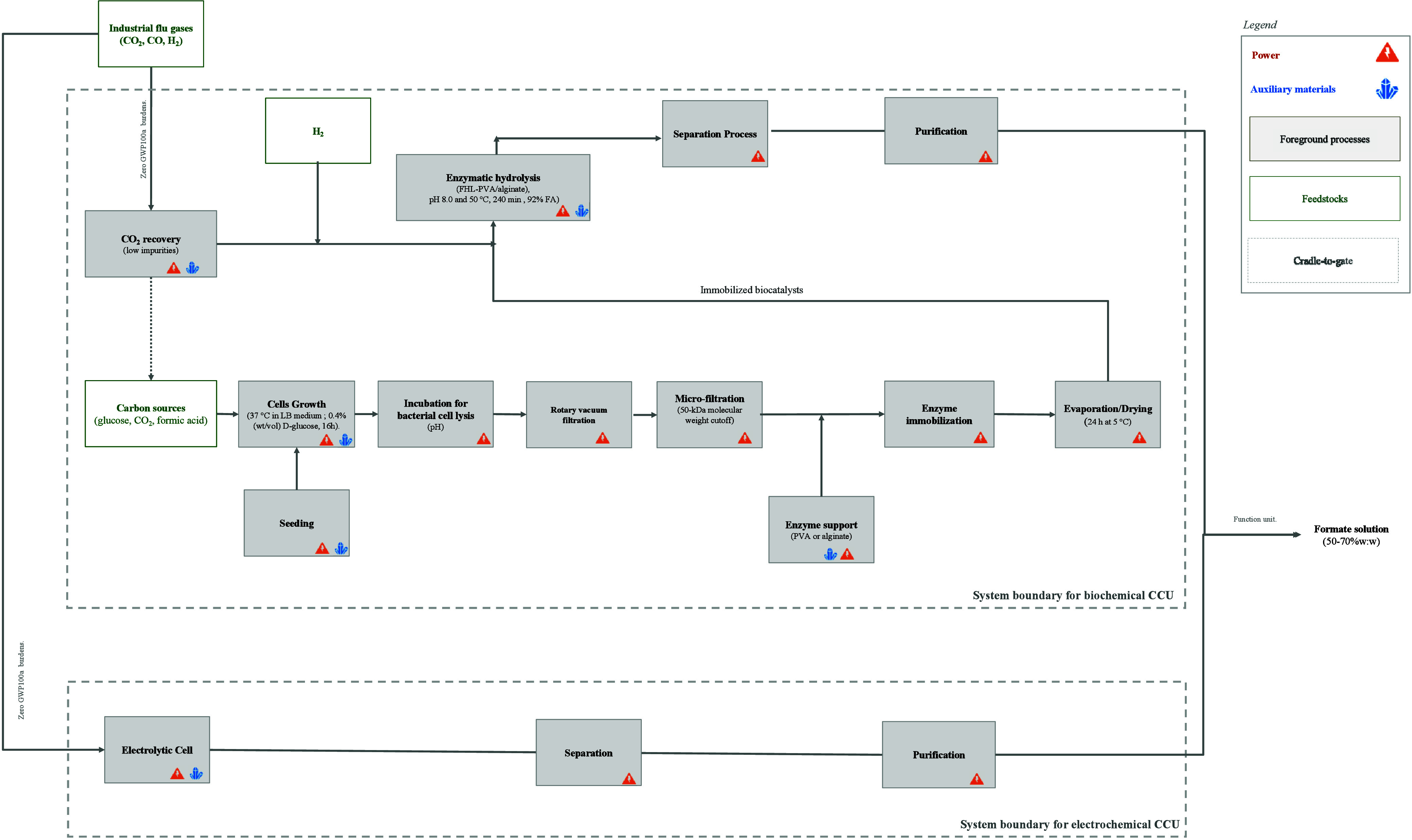
Block diagram and system boundaries.

### 3.2 Foreground Inventory

#### 3.2.1 Upstream Processes

For the biocatalytic pathway, CO_2_ from BFG was captured by an adsorption system with the CANSOLV technology due to its high TRL (≅9)^53^ and feasibility of industrial application (capture of up to 99% of the CO_2_ from post-combustion gases under pressurized absorption).^53^ For modelling the absorption of CO_2_, into a 2.50 M amine-based solvent^54^ (N-solvent), the Henry Coefficient was estimated by 1.50 kPa.m^3^/mol at 40 °C. Pure CO_2_ was used in the PBRs to avoid low yields due to deactivation of enzymes in the presence of gaseous impurities.^6,8^

For the electrochemical factory, CO_2_ from the industrial flue gas was absorbed directly in carbonate solutions to provide the electrolyte.^23,55^ Although, depending on the catalyst, a high KHCO_3_ concentration can favor formate generation, the electrolyzer operated under 0.10% wt CO_3_^2–^ HCO_3_^–^, replicating previous operational conditions for electrolysis.^56^ Inefficiencies due exchanging sodium and potassium ions were neglected.^23^

#### 3.2.2 Biocatalytic Route

The hydrogenation of CO_2_ occurred in pressurized PBRs with immobilized enzymes. The PBRs operated at P = 10 bar, yield of 500 mmol/L, and up to 98% efficiency on CO_2_ consumption.^18^ For accounting the impact of enzyme’s reposition, the production of enzymes by *Escherichia coli* with glucose^57^ and formate (100% CCU technology)^19^ was considered. To achieve 35 g/L dried cells in the fermentation broth (∼OD_600_ = 150, 1.0 OD_600_ = 0.3 g/L dried weigh cells for *E. coli*),^57^ the microbial growth considered the experimental ratio of 50 OD_600_:150 mM glucose, leading to, approximately, 0.55 g of dried cells per 1 g of substrate.^57^ The protein mass fraction associated with the enzymatic sector was approximated to be 30%.^58^ Whereas the growth with formate/formic acid and CO_2_ as carbon sources^20^ considered the ratio of gas per mol of bacteria cells equal to 10 mol for CO_2_, 18 mol for O_2_, and 72 mol for other gases (pH = 7.0, 32 °C, 1 bar).^19^ To produce 1.0 g/L cells, 0.055 g/L FA was required.^19^ After extraction (10 mg of protein/mL enzyme)^15^ and concentration, the enzyme complex was immobilized in PVA (E_PVA_) or calcium alginate (*E*_a_)^15^ supports. The operational conditions are displayed in Table 2.

**Table 2:** Key Operational Conditions for BR^15,19,20^

**Carbon source**		
**CO**_**2**_ **+ Formate**	**Glucose**	**Operational conditions**
471.38 (28.00% wt)	734.11 (84.40% wt)	ton enzyme (protein)/year
50.00	50.00	% PVA recycle
1353.62	842.78	m^3^/year
4.48	4.48	ton of enzyme/reactor (∼17 mg/mL, Vr ∼ 270 m^3^)
17.90	17.90	ton of enzyme/factory cycle
4.00	4.00	reactors
24.00	24.00	h of reaction/hydraulic retention time
13	8	cycles without losing activity (1 cycles = 1 day ∼ 24 h of HRT/reaction)
338.40	210.70	m^3^/reactor/year
26.96	26.14	m^3^/reactor/cycle < Vr ∼ 270 m^3^/reactor
301.00	193.00	operating hours of the enzymes without losing activity
26	41	enzyme replacement/year

#### 3.2.3 Electrocatalytic Route

The catholyte operated saturated with CO_2_ and KHCO_3_.^23^ The anolyte operated with glycerol and an aqueous alkaline solution. The scenarios consider the production of sodium and potassium formate.^23^ Experimental productivity of 0.024 M/h (= 1.08 g L^–1^ h^–1^, 1.2 M in 50 h^23^) was assumed, which led to ∼30% of the theoretical yield (HCO_2_ Na/CO_2__(g)_ ≅ 1.023 *w*:*w*). Salt precipitation was neglected by assuming that the designing of electrolysis in industrial conditions kept the concentration of (bi)­carbonates under solubility targets (S_NaHCO_3__ = 1.40 M and S_KHCO_3__ = 2.24 M at 20 °C).^59^ The operational condition was displayed in Table 3.

**Table 3:** Key Operational Conditions for ER^23^

System specification	Target value/ranges of operation
Yield _Max._	1.2 M (after 50 h)
*CO*_2_ flow rate^a^	0.3 L min ^–1^ ∼ 0.6 g min ^–1^
Faradaic efficiency	81%
Constant current	100 mA cm^–2^
Potential applied^b^	5 V
Recirculation of salts or base[a]	
Catholyte	0.4 L min ^–1^
	0.1 M KHCO_3_ / K_2_CO_3_
Anolyte	0.8 L min ^–1^
	(3 M KOH/NaOH; 0.1 M of glycerol)

a Flow rates ratio (L/min:L/min): 1 CO_2_:1.380 catholyte:2.527 anolyte.

b The power demand of this system can vary in the range of 1.36–7.92 kW/(formate kg/h). The energy demand was fixed as, approximately, 7.7 kW/ (formate kg/h), as further described in the SI.

Hydrogen and oxygen evolution^60^ was considered in the cathode and anode chambers, respectively. The generation of other byproducts was neglected. Since the equipment design was used only to forecast capital costs, the number of stacks, for instance, was not estimated.

#### 3.2.4 Downstream Processes

The downstream process consisted of separating/recycling and purifying the final product. De-gasification was used to recycle the gas flows or just remove them from the aqueous solutions. The electrodialyzer recovered the formate in an aqueous solution and recycled the auxiliary materials. In both systems, the bipolar membrane electrodialyzer^61,62^ also pre-concentrated the formate solution to reduce the steam demand on the multi-effect evaporator. The maximum stacks for the electrodialyzer were fixed in 600. The multi-effect evaporation was used to obtain the final grade of formate salts (50–70% wt).

Other assumptions, mass flows, energy demand, and a process diagram from SuperPro Process Design, are provided as SI.

### 3.3 Environmental Modelling

Brightway2 ^63^ software, Ecoinvent (attributional, cutoff^64^),^65^ and PREMISE^35^ databases were used for LCA. Mid-point impacts were assessed using the Environmental Footprint (EF v3.0) characterization factors.^66^ The carbon capture of each technology was calculated based on the CO_2_ consumption and offset from the factory’s emissions.^50^ The allocation of BFG burdens was allocated to the polluter to avoid double counting: polluter’s pays principal. The background process considered Europe and UK data, as listed in the SI.

The prospective scenarios for 2040 emissions comply with the climate change mitigation target of Paris Agreement objectives by considering efficiency improvements, renewable energy electrification, and other decarbonization strategies.^35^ Extrapolation from historical developments was used to forecast the embodied carbon of the background processes by assuming climate change mitigation targets of SSP2-RCP2.6 scenario–equivalent to an atmospheric temperature increase below 1.6–1.8 °C (GMST) by 2100 with respect to the pre-industrial levels.

### 3.4 Techno-Economic Modelling

NPV, revenue, OpEx, and TCI were used to compare the technology’s feasibility. The software SuperPro Process Design was used to design the mass-energy balance and perform the economic analysis for the 2024 landscape with USD currency. The construction period was 30 months, with a startup period of 4 months. The project’s lifetime was projected to be 30 years, with an internal rate of return set at 7% by default with the inflation rate at 4%. Catalyst cost was overhead, if not specified. Supplementary cash flow information and unit costs are provided as SI.

### 3.5 Sensitivity and Uncertainty Analyses

Global sensitivity analysis was used to forecast uncertainties in ENaF70% and BNaF70% (CCU and G) scenarios. The Sobol sampler^67^ generated 1 × 10^+4^ random points between a fixed range of uncertainty. The probability density (kde) was acquired by considering variabilities in both the inventory and costs.

Foreground uncertainty assumed ±30% of variation on all inputs/outputs flows and utilities, except for the productivity (±5%, manufacturing variation only), electrolyte, and H_2_. The H_2_ consumption was fixed in the inventory to forecast only the impact of its supply since benefits of greener technologies might be relevant. Then, PCF_H_2__ was assumed to vary from 0.20 kg of CO_2eq_/kg^68^ up to the default emissions of the database. For electrolyte replenishment, the variation from 0.20% up to 1.00% (default) of total utilization was analyzed—if potential process’ optimization with experimental data could mitigate losses.

The unit cost uncertainty was the synergy between inventory and cost variability. The electricity/power expenses fluctuated from 0.07 ^10,11^ to 0.10 USD/kWh. The H_2_ purchase cost varied between 0.60–1.0 USD/kg.^69^ Other OpEx varied only with the foreground range. Facility dependent costs were excluded.

## 4 Conclusion

The successful deployment of CCUt for industrial decarbonization requires a thorough understanding of supply chain complexity and LCA. In terms of technology, the average unit cost of formate with different grades was lower for electrocatalysis ($1.07 to $0.71/kg) than for biocatalysis ($1.39 to $0.93/kg). Uncertainties applied for formate 70% showed average unit costs of 1.25, 1.50, and 1.60 for ENaF70%, BNaF70%G, and BNaF70%_CCU_, respectively. The CapEx of equipment reached 30% of TCI. In biocatalysis, capturing and compressing equipment represented 39% of the total costs. On the other hand, electrolysis dominated electrocatalysis CapEx (89%). The power supply was a predominant cost in both factories, exceeding 60% of the total OpEx. The use of alkaline solutions was crucial in both technologies, with electrolyte replenishment being a key cost driver only for ER. In BR, H_2_ consumption was also substantial. Charging for the CO_2_ treatment generated sufficient revenue to achieve profitability in both pathways, which could ignite the deployment of CCU at the current TRL. Additionally, depending on the final application, lowering the final product grade demonstrated economic benefits, creating profitable scenarios for both technologies. The overall life cycle of formate for biocatalytic route led to better environmental performance than electrocatalysis. UA indicated a 58% probability of biocatalysis achieving a lower PCF than the baseline, compared to 2% for electrocatalysis. Energy demand, electrolyte replenishment, and H_2_ consumption were key contributors to emission’s variability. To mitigate formate’s PCF with the current TRL of BR and ER, the supply chain shall be designed to support low-carbon energy grid and H_2_ flows.

CCUt development for future deployment must focus on optimizing operations, supply chain design, and policy strategies. Governments should ensure long-term resource availability to prevent CCU from becoming a short-term or costly solution. Without this planning, CCUs could become an expensive investment for stakeholders and may only serve as a short-term solution due to a lack of carbon resources needed to operate in a net zero economy. Additionally, simple transportation or pipeline systems for feedstocks (CO_2_ and H_2_) are required for effective CCU strategies. Future priorities include: 1) R&D investments to enhance efficiency; 2) resource management of the supply chain, including infrastructure and affordable green energy/feedstocks; 3) creating a new market for industrial flue gases; and 4) potentially considering low-grade uses.

## Supplementary Material



## Data Availability

Additional data that support the findings in this study are available upon reasonable request to the corresponding authors.

## Notes

State-of-the-art review, describes details about the modelling, including assumptions for TEA and LCA inventory, and briefly discusses the OpEx in different regions for each technology, and additional references 70–115 (PDF)

## LIST OF ABBREVIATIONS

ACP [mol H^+^_eq_/FU]: Acidification potential
ADP [kg Sb_eq_/FU]: Abiotic depletion potential
BFG: Blast furnace gas from steel manufacturing (industrial flue gas)
BM: Bipolar membrane
BR: Biochemical route/CO_2_ and H_2_ conversion into formate by biocatalysis (enzyme: formate hydrogenlyase)
CapEx: Capital expenditures
CCU: Carbon capture and utilization/use
CCUS: Carbon capture use and storage
CCUt: Carbon capture and use technology
*E*_a_: Enzymes immobilized in alginate
EB: Biochemical route
E_PVA_: Enzymes immobilized in PVA
ER: Electrochemical route/CO_2_ electroreduction
ETS: Emission trading schemes
EU: European Union
FDL: Formate dehydrogenlyase
FEP [kg P eq/FU]: Freshwater Eutrophication potential
FHL: Formate hydrogenlyase-1
FTP [CTUe/FU]: Freshwater Ecotoxicity potential
FU: Functional unit (1 kg of MP, aqueous solution of formate)
G: Enzymes production from microorganism growth in glucose (G) broth
GWP100a: Global warming potential for 100-year time horizon (or PCF)
KF: Potassium formate
LCA: Life cycle assessment
LUP [-/FU]: Land use potential
MEP [kg N eq/FU]: Marine Eutrophication potential
MP: Main product (sodium or potassium formate)
NaF: Sodium formate
NPV: Net present value
N-solvent: H_2_O and *N*-methyldiethanolamine (70:30% wt)
ODP [kg CFC-11_eq_/FU]: Ozone depletion potential
OpEx: Operational expenditures
PBRs: Packed bed reactors
PCF [kg CO_2eq_/FU]: Global warming potential
PCF: Product carbon footprint (PCF)
PVA: Poly­(vinyl alcohol)
SA: Global sensitivity analysis
TCI: Total capital of investment
TEA: Techno-economic analysis
TEP [mol N eq/FU]: Terrestrial Eutrophication potential
TRL: Technology readiness level
UA: Uncertainty analysis
UK: United Kingdom
USA: United States of America
WUP [kg world eq deprived/FU]: Water use potential
+: Additional revenue related to BFG
