# Supplementary material for: Carbon Capture and Utilization for Sustainable Supply Chain Design of Intermediate Chemicals: The Formate Factory
Source: ACS Sustain Resour Manag. 2025 Mar 7;2(5):733–43. doi: 10.1021/acssusresmgt.4c00472 (PMC12105007; doi:10.1021/acssusresmgt.4c00472)
Supplement: Supplementary file 1 [file rm4c00472_si_001.pdf]

Carbon capture and utilisation for sustainable supply chain design of intermediate chemicals: the formate factory

SUPPLEMENTARY MATERIAL

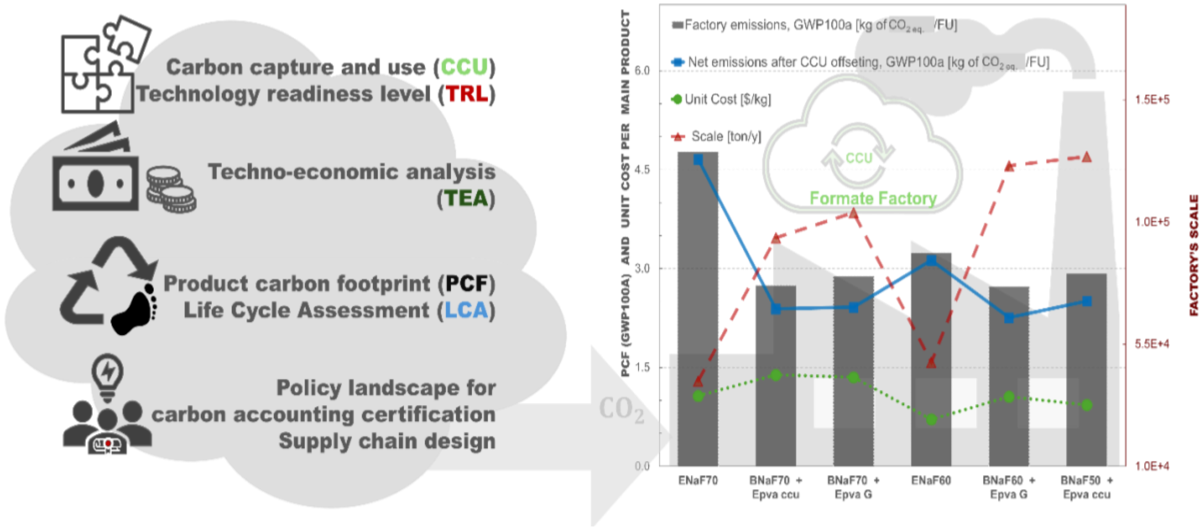

Ariane Silveira Sbrice Pinto [a] †*, Nalan Gulpinar [a], Fang Liu[a], Elizabeth Gibson[b], Linsey Fuller[c], and Philip Souter[c].

[a] Business School, Management Department, Durham University, DH1 3LB, Durham, England, United Kingdom.

[b] School of Natural and Environmental Science, Newcastle University, NE1 7RU, Newcastle-upon-Tyne, England, United Kingdom.

[c] Procter and Gamble, Newcastle Innovation Centre, Whitley Road, Longbenton, Newcastle upon Tyne NE12 9TS, England, United Kingdom

† Procter and Gamble, Newcastle Innovation Centre, Whitley Road, Longbenton, Newcastle upon Tyne NE12 9TS, England, United Kingdom.

* First author.

E-mail: sbrice.a@pg.com. Phone: +44 0191.228.1000

SUMMARY

1. Inventory....................2

1.1. State of the art....................2

1.2. Foreground process....................5

1.2.1. Upstream process: absorption-desorption....................5

1.2.2. Biocatalysis....................8

1.2.3. Electrocatalysis....................12

1.2.4. Downstream....................16

1.2.5. Other considerations....................16

1.3. Background process....................21

2. Techno-economic inventory....................21

3. Extra results....................29

4. Discussion of economic uncertainty....................29

REFERENCES....................*36*

1. **Inventory**

1.1. State of the art

Biochemical technologies use microorganisms [14,18] or enzyme complexes[8] to catalyse CO_2 fixation into formate under mild conditions. The interplay between FDHs and FHL complexes enables the interconversion of H_2, CO_2, and formate in bacteria. In *Escherichia coli*, for example, the FHL-1 enzyme complex functions under fermentative conditions, coupling FDH and hydrogenase components. Under high-pressure fermentation (P > 2 bar), *Escherichia coli* has demonstrated formate production of up to 500 $mmol \cdot L^{-1}$ [14,18]. Pressurised reactors improve gaseous feedstock solubility [14,18], mitigating mass-transfer limitations associated with CO_2 capture, and enhancing yield [14,18]. Despite promising results of fermentation technology, several bottlenecks remain for large-scale implementation: a) substrate solubility, leading to gas-liquid mass transfer limitations and rapid H_2 escape; b) by-product formation, as acetate, lactate, and succinate [14] , which lead to a complex recovery of the MP in downstream processes.

To overcome challenges of by-product formation, the biocatalytic route has been studied with FHL / FDH enzymes from *Escherichia coli* [13,70]. The biocatalysis minimises by-product formation by leveraging enzyme specificity. Encapsulation offers additional advantages[16,71], such as reducing replenishment costs of biocatalysts [15] or microorganisms[16,17] over multiple reaction cycles, facilitating separation from liquid solutions, and ensuring stability in continuous operations [16,17]. Field tests have achieved a product concentration of 18 mM within 48 hours under atmospheric conditions (pH = 6.5, T = 30°C), using immobilised FDH and other oxygen-tolerant enzymes capable of converting CO and CO_2 into formate [6,8].

Beyond the former advantages, *Escherichia coli* has been engineered to grow using solely in formate - CO_2[19,20] as carbon source. This can be a groundbreaking opportunity for further decarbonisation in a circular economy since the carbon footprint associated with glucose-based bacterial growth could be avoided. In this context, the biocatalytic production

of formate holds significant potential for the sustainable development of CCU industries by utilising CO_2 as the sole carbon source while minimising by-product formation. However, despite its industrial potential, the use of immobilised enzymes to convert flue gases into formate remains at the proof-of-concept stage, requiring further insights into the techno-economic and environmental feasibility of large-scale production.

Electrochemical technologies, instead, directly reduce CO_2 into formate pairing metal-based catalysts electron transferring[9]. Extensive research has been conducted to optimise these systems, with pilot tests demonstrating faradaic efficiencies exceeding 80% at high current densities (up to 1 A cm^{-2}). Electrochemical performance varies based on reactor design, electrolyte composition, faradaic efficiency (50–100%), current density (0.421–1 A/cm^2), pH (alkaline/neutral), and cell voltage (0.6–5 V RHE) [22] .

Despite these advancements, challenges remain for large-scale implementation, including energy losses, catalyst deactivation [72], side reactions [73] (such as by-product formation and oxygen/hydrogen evolution reactions [60]), and salt precipitation. The highest formate production rates have been observed when using (bi)carbonates (K_2CO_3/$KHCO_3$) as catholytes [22], due to their role in supplying carbonaceous reactants for electroreduction. The integration of (bi)carbonate-based electrolysis within CCU factories using BFG presents a significant advantage, as it simplifies system design by allowing direct dissolution of industrial flue gases into carbonate solutions, eliminating the need for a separate CO_2 capture stage [22,56]. Performance data from flow-cell electrolysis using 3.0 M $KHCO_3$(aq) indicate nearly comparable results to gas-fed electrolysers, achieving 64% faradaic efficiency at 4 V, pH = 10, and 100 mA cm^{-2} [74]. Formate concentrations of up to 1.2 M have been obtained within 50 hours at the cathode chamber using aqueous (bi)carbonate solutions [23]. Recent developments have further advanced the scalability of electrochemical formate production [24], with electrochemical

stacks reaching active areas of up to 40,000 cm^2, while maintaining catalytic stability at current densities of 1 Acm^{-2} [23,24].

Compared to biochemical methods, electroreduction technology demonstrates a higher TRL, with pilot-scale trials converting 146 kg of CO_2 per day into 110 kg of formate/formic acid per day [22]. In contrast, biocatalytic reactors using immobilised enzymes and industrial flue gases as feedstock achieved production rates below 1 kg of formate per day [8]. While electrochemical technologies offer superior productivity, their industrial-scale deployment comes at the cost of high energy demands, which may affect overall CCU factory performance compared to the relatively low energy requirements of biochemical technologies. Future research should further investigate the trade-offs between these two approaches, particularly in the context of achieving net-zero targets and supply chain design.

Indeed, the widespread adoption of formate production depends on integrating CCU technologies into existing industrial supply chains while addressing operational costs, carbon accounting frameworks, and regulatory requirements. Life cycle assessments have demonstrated that formate derived from CO_2 can reduce over 80% of the PCF [25]. However, economic feasibility remains a challenge due to low yields, high consumption of auxiliary materials (e.g., CO_2), and substantial energy demands for both the production and purification stages [10,11].

Economic assessments indicate that the profitability of CCU pathways is highly sensitive to process parameters. Electrocatalysis unit costs vary significantly, ranging from \$400 to \$2,095 per ton [75] —excluding expenses related to maintenance, depreciation, interest, and taxes. Mostly, key factors affecting profitability of producing chemicals from CO2 include electricity costs (<\$0.07 per kWh) and CO_2 commodity prices (<\$40 per ton) [10,11]. At the best of our knowledge, techno-economic-environmental aspects of producing formate with biocatalysis are still unclear. Additionally, comparative assessments of both technologies are

still missing, and further investigation must be conducted under consistent assumptions—such as pricing models and LCA's system boundaries—to ensure transparency in both economic and life cycle analyses [27,28].

1.2. Foreground process

The formate factory consists of upstream, main, and downstream processes. The upstream block included the feedstock preparation. In both CCU factories, blast furnace gas (BFG) from steel production was considered as feedstock due to its high availability in the UK(~900 tonne of BFG/h) and its concentration of CO_2 (~36%*wt* of CO_2) in its composition. Table 1 compares the BFG composition used in this work with syngas from biomass – another potential carbon source for chemicals production in CCU factories.

Table 1- Molar composition of industrial flue gases.

	CO	H_2	N_2	CO_2	CH_4	C_2H_6	H_2O	References
BFG	23.9	4.4	49.0	22.7	-	-	-	76
Syngas (Lignocellulosic materials)		30-60	25-30	-	5-15	0-5	-	77

The default main product (MP) was an aqueous solution with 70% of formate. The variation in the grade of the MP was analyzed in different scenarios.

1.2.1. Upstream process: absorption-desorption

Amine-based Solvent

The feedstock was pre-treated to capture the CO_2 and remove potential inhibitory /deactivators compounds. The CANSOLV process [78] was indicated by P-2 in Fig.2-a. The selection of CANSOLV process considered its high technology readiness level and feasibility

of industrial application [53]. Combined tertiary amines in water solution captures CO2 in this process. The organic solvent was recovered in a stripping column (P-3). Here, piperazine was used as a reference from the blend of tertiary amines since SuperPro® database contains all its physical-chemical properties for thermodynamic estimates ($C_4H_{10}N_2$, , MW=86 g/mol – the single solvent used to estimate the mixed of tertiary amines in the CONSOLV solvent). Although the reaction between piperazine and CO2 is complex since different intermediates can be produced in their protonated (or not) forms[79] , the capture efficiency of using the synergy between different tertiary amines in aqueous solutions was successfully proven with CANSOLV technology. Indeed, the absorption of CO2 could vary according to proportions of water, amine, and CO2 mixture. The absorption capacity of one specific amine solvent can vary from 0.90 to 1.26 mol CO2/mol single amine. The regeneration efficiency of amine solvents can be estimated in 74%. In MEA (30%wt), for instance, the common absorption capacity is lower than in CANSOLV solvents, reaching 0.46 mol of CO2/mol of MEA[80]. In the CANSOLV process the variation ranged from 15%wt -64%wt at 60oC and 1 bar was reported [78]. The CANSOLV patent reported the maximum absorption capacity of 64%wt for 60oC, 1 bar, and an aqueous solvent containing the total of 30%wt of amines [78]. Large scale application of CANSOLV CO2 capture system indicated the capture of up to 99% of the CO2 from post-combustion, low-pressure off-gases [78 53], indicating an attractive technology for CCU factories. Although CANSOLV shall require desulphurization of industrial flue gases that are rich in SO2 to mitigate the degradation of the solvent [78], the composition of BFG does not indicate the requirement of this additional process (Table 1).

The design of absorption columns in carbon capture facilities for industrial applications shall considerer diameters (D) in the range of 6m to 15 m and length/high to diameter (L/D) ratio should be less than 30 [81]. If necessary, the maximum D of towers was assumed to be 8 m

and all heights were limited to 60 m to avoid wind, load, and foundation concerns [81] while maximizing economic advantages of scaling up the manufacturing process.

The organic solvent was recovered by heating the CO2-rich solution in a flash (P-3). The Henry Coefficient of CO2 in the MEA-CO2-H2O System was equal to 1.5 kPa.m3/mol at 40oC (0.5-0.6 mol CO2/mol MEA, 2.5 M MEA) [54].

Carbonate-based Solvent

Novel carbonate-based CO_2 capture process with microbubble systems have been developed to capture post-combustion gases[82,83]. Here, the preparation of the catalytic solution for the electrochemical technology considered mixing of BFG with carbonate salts directly in P-3. CO_2 from the industrial flue gas was absorbed directly in carbonate solutions [23], according to the overall stoichiometry from Eq. (1) [55].

$$K_2CO_3 + H_2O + CO_2 \rightleftharpoons 2KHCO_3 \qquad \text{(A.0)}$$

Experimental data have shown variations from 10-40% () in sodium/potassium carbonates to bicarbonates conversion [56] due to mass transfer limitations. The maximum conversion of Na2CO3 (10% *w: w*) at 30 °C was 70% [55]. Although, depending on the catalyst, high KHCO3 concentration can favour formate generation, the electrolyser operated under 0.1% (*wt*) CO_3^{2-} /HCO_3^-, replicating previous operational conditions [56] . Experimental data have shown variation from 10-40 % (*wt*) of carbonates to bicarbonates conversion [56] due to mass transfer limitations.

The electrolyser operated with concentration below 0.1%(*wt*) CO_3^{2-} /HCO_3^{-}[56]. Here, the CCU factory estimated 20%(*wt*) of Na_2CO_3 to $NaHCO_3$ conversion. Although former boundary considered potassium carbonate kinetics [56], variations in Henry's constant of potassium and sodium carbonate were not meaningful [84] for aqueous solution with low

carbonate concentration (<1 M, T=50°C) [84]. Besides, the utilisation of both salts has been reported in the literature [23]. Inefficiencies due exchanging sodium and potassium carbonate was neglected and only the economic impact of the price of the salts were evaluated. The solubility of CO_2 in 5% (*wt*) carbonate solutions at 25°C varied from 0.016 (T=50°C) to 0.024 (T=25°C) mol L^{-1} bar^{-1} [56]. This range was used to limit the concentration of dissolved CO_2 in the catholyte. The BFG was used without additional purification and potential deactivation of the catalyst in the presence of gaseous impurities were neglected.

1.2.2. Biocatalysis

The CO_2 was mixed with the reaction medium (P-11; P-17) and sent to the PBR (P-10; P-19). The PBR was chosen to evaluate the potential production of formate with biocatalysts. Formate oxidation and CO_2 reduction with microbial pathway is an interconvertible process that is carried out by a combination of enzymes. The reactions involved were presented in Eq. (A.1) [85].

$$H_2 \leftrightarrow 2\,H^+ + 2e^- \qquad \text{(A.1-a)}$$
$$CO_2 + 2H^+ + 2e^- \leftrightarrow HCOOH \qquad \text{(A.1-b)}$$

The overall reaction was

$$H_2 + CO_2 \underset{XOH}{\overset{}{\underset{FHL}{\Longleftrightarrow}}} HCOOH \qquad \text{(A.1)}$$

The term ‘formate hydrogenlyase’ can be used in general terms for any disproportionation of formate into CO_2 and H_2 by any combinations of different enzymes. The formate hydrogenlyase-1 (FHL) is a complex of enzymes that is found in gram-negative bacteria.

FHL from *E. coli* was highly sensitive to changes in extracellular pH, carbon source (water-dissolved CO_2), and nutrients composition [86]. Here, a high-pressure system was used to

provide high CO_2 concentrations in the liquid phase during the biocatalytic reaction [14,18]. As discussed, high purity CO_2 was used since *E. coli* native hydrogenase was sensitive to O_2 [87] and CO [70]. Formate concentrations can vary with the system's pressure from 85 (2 bar) to 500 (10 bar) mmol/L (37°C, pH=8, 44% H_2: 56% CO_2, *wt*) [18]. The default value in the biochemical factory was the maximum yield. The highest efficiency of 98% for CO_2 consumption [18] was reported for free *E. coli* [14,18] technologies. However, the potential application of immobilized enzymes is promising for industrial systems due to its specificity and easy recovery of the final product.

The enzymatic pathway was efficient for catalysts extracted from *Candida boidinii* under atmospheric pressure, where the immobilization in polyvinyl acetate (PVA) resulted in both thermal stability and 12% efficiency increase (80% for free and 92.50 % for immobilized enzymes) compared to free enzymes without significative activity loss for up to 4 cycles [15]. Although experimental validation of this route is still not available for *E. coli*, the modelling of the potential PBR system could help to understand the capacity of CO_2 capture into formate and techno-economic opportunities/ bottlenecks of this technology deployment. Material requirements for producing and immobilizing enzymes and its costs were initially neglected, then scenarios that considered the production- reposition over time of immobilized enzymes were evaluated.

Escherichia coli can be grown aerobically by using glucose [21] or formic acid-CO_2 [19,20] as carbon source. The utilization of formate as carbon source followed the proportionality of the glucose process (Fig. 2-c and -d). The potential growth of *E. coli* on CO_2 and formic acid alone [19] was explored here as a potential route to produce formate using exclusively CO_2 as carbon source- a 100% CCU factory. The former approaches are innovative since they forecasted the potential use of biochemical technologies for producing intermediate chemicals with 100% of CCU. The utilization of glucose [14] during the bacteria growth was used as

reference and prospective scenarios considering formic acid/formate and CO_2 as carbon sources [19,20] were forecasted. Besides, peculiarities of co-producing acetate (16.7% *wt*), formate (32.5% *wt*), lactate (30% *wt*), and succinate (3.3%*wt*) were not modelled [14].

Glucose, ammonia, and other medium components are converted into an industrial enzyme in a fed-batch, aerobic culture with aeration rate of 0.85 VVM (volumes of air per volume of liquid per minute) [57]. The production of the bacteria included lab-screening in eleymeyer flasks, seeding (P-42; P-43; P-47), growth, and enzymes production (P-45). To achieve 35 g/L of dried cells (~OD_{600}=150, 1.0OD_{600}=0.3 g/L of dried weigh cells for *E. coli* [57] in the broth of fermentors, the microbial growth considered the experimental ratio of 50 OD_{600}:150mM glucose, leading to , approximately, 0.55 g of dried cells per 1 g of substrate [57]. Considering glucose (G) and only salts and nitrogen (NH_4OH) as micro-/macro- nutrients, respectively, the stoichiometry (mass basis) of bacteria growth (pH=7, 30°C, 1 bar) was estimated with Equation (A.2).

$$100\ \mathrm{G} + 10\ \mathrm{NH_4OH} + 50\ \mathrm{O_2} + 2\ \mathrm{Salts} \rightarrow 50\ \mathrm{Biomass} + 50\ \mathrm{CO_2} + 62\ \mathrm{H_2O} \quad \text{(A.2)}$$

The protein mass fraction associated to enzymatic sector (fraction ϕ_E) was approximated to be 30% [58].

Whereas the growth with formate and CO_2 as carbon sources [20]considered excess of gases to achieve the desired production. The quantity of gases provided were 10 mol of CO_2, 18 mol of O_2, and 72 mol of other gases per mol of bacteria cells (pH=7, 32°C, 1 bar)[19] . To produce 1 g/L of cells, 0.0552 g/L of formic acid (FA) was required [19]. Then, the mass stoichiometric for cell's growth was estimated with Equation (A.3).

$$3\ \mathrm{FA} + 95\ \mathrm{CO_2} + 9.5\ \mathrm{NH_4OH} + 137\ \mathrm{O_2} + 1\ \mathrm{Salts} \rightarrow 50\ \mathrm{Biomass} + 15\ \mathrm{Enzyme} + 180\ \mathrm{H_2O} \quad \text{(A.3)}$$

Both fermentation procedures occurred in bath- reactors (P-048, V~150 m^3) at atmospheric conditions. At the end of the fermentation, the broth was cooled to 12 °C to avoid

deactivation and the bacteria's cell wall was broken by changing the pH (the amount of acidic solution was neglected) . The biomass was separated from the medium in a rotary drum filter (P-49). The enzymes were recovered by micro-(P-51) and ultra-(P-52) filtration prior to the immobilization phase. The enzyme content of the extract was estimated to be, at least, 10 mg of protein/mL of enzyme [15].

The immobilisation of enzymes can be done through a variety of methods. Here, two enzyme supports were analysed: PVA[39] and calcium alginate [39] . The formulation of the beds occurred in CSTRs (P-56; P-58). The recovery of the immobilized enzymes (P-62) varied according to the polymer. The PVA catalysts were recovered in a spray-dryer and the alginate beds were recovered by a rotary drum filter. **Error! Reference source not found.** summarised the operational conditions of the enzymes production. Fig. 2-a, -c, and -d showed the biochemical factory, the production of enzymes and its encapsulation with alginate and PVA, respectively.

Table 2 – Key-operational conditions for the formate production trough profitable biocatalytic routes [15,19,20].

**$B_{NaF\ 50\%}$ E_{pva}**	**$B_{NaF\ 50\%}$ E_{pva} G**	**Operational conditions**
471.38 (28%% *wt*)	734.11 (84.40% *wt*)	ton E (protein) /y
50	50	% PVA recycle
1353.62	842.78	m3/y
4.48	4.48	ton of E /reactor (~17 mg/mL, Vr~270m3)
17.9	17.9	ton of E/ factory cycle
4	4	Reactors
24	24	h of reaction/ HRT
13	8	cycles without losing activity (1 cycles=1 day~24h of HRT/reaction)
338.40	210.70	m3/reactor/y
26.96	26.14	m3/reactor/cycle <Vr~270 m3/ reactor
301	193	operating hours of the enzymes without losing activity
26	41	enzyme replacement /year

Both PVA and alginate beads can be recycled [39,88], however, the economic benefit of recycling was only considered for PVA beds. This assumption was made due to its high costs. Although this technology has low TRL (≅ 3, proof of concept [89]) for FHL from *E. coli*, the modelling of the potential PBR system could help to understand the capacity of CO_2 capture into formate and techno-economic opportunities/ bottlenecks of the technology deployment for future research and development investments.

Limitations of the biochemical approach include small thermodynamic driving force under low substrate and high product concentrations[86] The costs of the PBRs were underestimated since the design of plug-flow reactors (PFRs) was used to forecast their CapEx in SuperPro Design®. Here, it was important to highlight that size-limit was assumed to adapt to realistic PBRs. The resolution of the glucose and 100% CCU models were not similar since production of enzymes (*E. coli*) with CO_2 -formate was still under development and the lack of data hampered the robustness of this model, while the glucose as carbon source is a well-known bioreaction. Another significant point was the stability of the enzyme's capsules over time, which was difficult to guarantee with lab-scale data. The experimental validation of the amount of protein was also a limitation for the estimates.

1.2.3. Electrocatalysis

Formate/formic acid from CO_2 was produced in an electrocatalytic cell (P-1) in the CCU system and recovered as aqueous solution with 50 to 70% grade. The TRL was rated as 4 [89] . The source of NaOH/KOH was the anolyte chamber to control the pH (P-2). The catholyte was a bicarbonate/carbonate buffer saturated with CO_2 from the industrial flue gas (P-3) [23], as described in upstream section. The electrolyser operated with concentration below 0.1%(*wt*) $CO_3{}^{-2}$ - $HCO_3{}^{-}$[56]. Although the presence of H_2/CO in the BFG could gradually decrease the

electrolyser productivity over time in formate electrolysers (e.g., up to 20% in 20h for Pd on activated carbon[72]), these inefficiencies were neglected in all scenarios of our study.

In the electrolytic cell (P-1/EC), the half equation of formic acid production was shown in Eq. (A.3).

$$CO_2 + 2H^+ + 2e^- \rightarrow CO_2H_2 \qquad [E^O = -0.20 \text{ V vs RHE}] \qquad \text{(A.3)}$$

The productivity of formate/ formic acid ion the electrolyser reached, approximately, the productivity of 0.024M/h (1.2 M in 50h [23]), which led to ~30% of the theoretical yield ($CO_2H_2/CO_{2\,(g)} \cong 1.046, wt$).

The oxygen evolution reaction (OER) was identified as a common reaction in the anodic compartment (P-02) across various reactor configurations for the continuous electroreduction of CO_2 to $HCOO^-$ and HCOOH [73]. OER is generally perceived as a sequence of single electron/proton charge transfer reactions [60]. The hydrogen and oxygen evolution [60] reactions (OER and HER, respectively) were given by equations (A.4-a and -b, respectively). In alkaline conditions for a RHE scale, for OER (anode)

$$2OH^-_{(aq.)} \rightarrow 1/2\ O_{2(g)} + 1\ H_2O_{(l)} + 2e^- \quad [E^O = 0.44 \text{ V vs RHE}] \qquad \text{(A.4-a)}$$

For HER (cathode),

$$2\ H_2O_{(l)} + 2e^- \rightarrow H_{2(g)} + 2OH^-_{(aq.)} \qquad [E^O = -0.83 \text{ V vs RHE}] \qquad \text{(A.4-b)}$$

The overall reaction was,

$$2\ H_2O_{(l)} \rightarrow O_{2(g)} + H_{2(g)} \qquad [E^O = -1.23\text{V}] \qquad \text{(A.4)}$$

To mitigate the hydrogen evolution reaction (HER), the electrochemical reduction of CO_2 in aqueous media is commonly conducted under alkaline conditions. The pH of the

electrolyser was controlled with NaOH/KOH. At pH ~3.45, the formic acid is predominantly dissociated into formate. The anolyte (P-02) solution was an aqueous mixture of NaOH/KOH (3M) and glycerol (polyol) [23]. Auxiliary chemicals demand [23], as polyol stabilizers (as hydrides, halides, phosphines, porphyrins) and/or metals (catalysts as Pb) was not calculated. The CCU technology was translated from data rated as 4 of Technology Readiness Level (TRL) scale [89].

The electrochemical cell assembly (membrane anion exchange, e.g.) followed the operational conditions indicated in Table 3 [23], which summarizes the reported data from the second example reported by Van Den Bosch's patent [23].

Table 3 –Operational conditions of the electrolyser [23].

**System specification**	**Target value / ranges of operation**
Yield (max.)	1.2 M (after 50h)~0.024 M/h
CO_2 flowrate *	300 mL/min~0.558 g/min.
Reposition**	0.273 mL/min<br>(20% glycerol: 80% of H_2O)
FE	81%
Constant current	100 mA/cm^2 ***
Potential applied	5V
Recirculation of salts or base	
Catholyte	0.414 L/min<br>0.1 M of $KHCO_3$ ($KHCO_3$ / K_2CO_3)
Anolyte	0.758 L/min<br>(3 M KOH/NaOH; 0.10M of glycerol)

* Gas diffusion electrode (GDE) and filter-press/three compartment electrochemical cell.
**The concentration of glycerol inside the cell might be 0.045-0.1M, so this solution was added during the process. The reposition of glycerol and water considered the degradation of the organic compound during the reaction. However, the simulations did not include this degradation, prioritizing the composition of the inputs (anolyte (S-103) and catholyte (S-101)) of the electrolytic cell (EC-101). The reposition flowrate was used to estimate losses. Flowrates ratio (L/min:L/min) : 1 CO_2: 1.38 Catholyte: 2.53 Anolyte (example2 from the reference).

The power demand was estimated in, approximately, 7.7kW-h/kg of formate., which was calculated by Eq. (A.5) and (A.6).

$$Power = \frac{E.i}{F_{formate}} \quad (A.5)$$

Where E is the applied potential (=5V, as indicated in Table 4), i is the total current, and $F_{formate}$ is the flowrate of formate in the electrolytic cell. Considering 1 cm^2 of electrode as reference, the Faraday's Law (electrolysis) for a constant current is defined as

$$n = \frac{i.t}{F_C.v} \quad \text{Eq.(A.6-a)}$$

n is the number of mol of the main product (=mass/molecular mass), i is the current (Ampere), F_C is the Faraday Constant (96,485.3 C/mol), and v is the valance (mol of e-). Rearranging and considering the efficiency,

$$i = F_c.v\,\dot{n}/\varepsilon \quad \text{Eq.(A.6-b)}$$

where ε is the Faradaic efficiency for formate production.

Instead of using the area of the electrolyser to estimate its costs, the CapEx was estimated with the energy demand. Considering a membrane exchange technology for scaling up the electrolyser, the CapEx was estimated with an industrial cost of producing H_2 [90,91]. The former assumption was necessary since SuperPro Process Design (v.13) did not have electrolysers in its library. This estimate might slightly overcharge investments for the formate factory due to the inclusion of un-used equipments: compressors, gas reservoir, and the gas dryer. However, the TRL of producing formate is lower than manufacturing H_2, which might balance costs due additional expenses for designing of formate electrolyser.

Although the composition of industrial flue gases can impact on the efficiency of producing formate, this impact was out of the scope since limited information about the stability of the system was available to evaluate the impact of the gas composition, catalyst performance, and electrolyser productivity. Then, efficiency loss or additional OpEx related to

short lifetime of the catalyst due to deactivation was not considered. In an industrial context, it is expected a lifetime of 4 years for an electrode (35,040 hours), which requires testing 7,000 longer. However, electrolysers have been tested for short time scales (<200 h).

1.2.4. Downstream

The downstream consisted in purification of the final product. De-gasification (P-13; P-18; P-24) was used to remove gases from the aqueous solution before separating the formate from the catholyte. The electrodialyser (P-13; P-8) recovered the formate in an aqueous solution and recycled the auxiliary materials. In both systems, the bipolar membrane (BM) electrodialyser [61,62] also pre-concentrated the formate solution to reduce the steam demand on the multi-effect evaporator. The CapEx of the electrodialyser (P-8) was driven by the number of stacks of the equipment. The industrial process could consider more than 600 stacks. The higher the number of the stacks, the higher the CapEx, indicating that future design of this equipment could be essential to recover the product. Besides, the electrodialysis could be used to separate the final salts in formic acid plants, for instance. Additional experimental data could indicate the best membrane for this process. The hight selectivity in ED membranes will be essential to recycle electrolytes, avoiding extra costs with their reposition. Finally, the multi-effect evaporation (P-4; P-22) was used to obtain the final grade of formate (50-70% *wt*).

1.2.5. Other considerations

Since the development of the selected technologies was in different phases, the TRL was indicated according to Buchner *et al.* [89]. The concentration of gases in the liquid phase was calculated by considering Henry's law using gas constants at 298 K (~25°C) to be 1,282.1 $L.atm.mol^{-1}$ and 29.4 $L.atm.mol^{-1}$ for H_2 and CO_2 [18]. Phosphate salts and pH control with potassium and sodium cations were considered [15]. Since process control of the pH will be automated in a large-scale plant, the respective OpEx will be minimal for both technologies.

Here, the simulations were designed to demand the minimal amount of the sodium/potassium hydroxide to keep the concentration of the experimental test used as reference.

The material and energy flow from this work was summarized in Table 4 , 5 and 6.

Table 4 - Electrochemical Factory.

**Material balance and Energy demand**	**[Unit]**	**EKF 70**		**EKF 50**		**ENaF 70**		**ENaF 60**	
**Flow**		**in**	**out**	**in**	**out**	**in**	**out**	**in**	**out**
Formate	[kg/h]	0.0E+00	3.7E+03	0.0E+00	3.7E+03	0.0E+00	3.7E+03	0.0E+00	3.7E+03
CO	[kg/h]	3.2E+04	3.2E+04	3.2E+04	3.2E+04	3.2E+04	3.2E+04	3.2E+04	3.2E+04
CO_2	[kg/h]	3.0E+04	2.9E+04	3.0E+04	2.9E+04	3.0E+04	2.9E+04	3.0E+04	2.9E+04
O_2	[kg/h]	0.0E+00	7.0E+02	0.0E+00	7.0E+02	0.0E+00	7.0E+02	0.0E+00	7.0E+02
H_2 (BFG)	[kg/h]	5.8E+03	5.8E+03	5.8E+03	5.8E+03	5.8E+03	5.8E+03	5.8E+03	5.8E+03
Impurity (gaseous)	[kg/h]	6.5E+04	6.5E+04	6.5E+04	6.5E+04	6.5E+04	6.5E+04	6.5E+04	6.5E+04
Glycerol	[kg/h]	3.1E+01	3.1E+01	3.1E+01	3.1E+01	3.1E+01	3.1E+01	3.1E+01	3.1E+01
Sodium/Potassiuum bicarbonate	[kg/h]	3.2E+03	6.4E+00	3.2E+03	6.4E+00	3.2E+03	6.4E+00	3.2E+03	6.4E+00
Sodium/Potassiuum hidroxide	[kg/h]	2.6E+02	3.8E+02	2.6E+02	3.8E+02	2.6E+02	3.8E+02	2.6E+02	3.8E+02
Water (process)	[kg/h]	1.0E+04	9.5E+03	1.0E+04	9.6E+03	1.0E+04	9.5E+03	1.0E+04	9.6E+03
Chilled Water	[kg/h]	5.5E+06	0.0E+00	5.3E+06	0.0E+00	5.5E+06	0.0E+00	5.4E+06	0.0E+00
Cooling Water	[kg/h]	3.4E+04	0.0E+00	1.5E+04	0.0E+00	3.4E+04	0.0E+00	2.7E+04	0.0E+00
Steam	[kg/h]	6.1E+04	0.0E+00	5.8E+04	0.0E+00	6.1E+04	0.0E+00	5.9E+04	0.0E+00
Aqueous waste	[kg/h]	0.0E+00	9.8E+02	0.0E+00	3.3E+02	0.0E+00	9.8E+02	0.0E+00	7.3E+02
CO (emisisons)	[kg/h]	0.0E+00	3.2E+04	0.0E+00	3.2E+04	0.0E+00	3.2E+04	0.0E+00	3.2E+04
CO_2 (emissions)	[kg/h]	0.0E+00	2.9E+04	0.0E+00	2.9E+04	0.0E+00	2.9E+04	0.0E+00	2.9E+04
Gaseous impurity (N_2)	[kg/h]	0.0E+00	6.5E+04	0.0E+00	6.5E+04	0.0E+00	6.5E+04	0.0E+00	6.5E+04
H_2 (emissions)	[kg/h]	0.0E+00	5.8E+03	0.0E+00	5.8E+03	0.0E+00	5.8E+03	0.0E+00	5.8E+03
O_2 (emissions)	[kg/h]	0.0E+00	7.0E+02	0.0E+00	7.0E+02	0.0E+00	7.0E+02	0.0E+00	7.0E+02
Utilities (total energy)	[kg/h]	6.8E+04	0.0E+00	1.9E+04	0.0E+00	6.8E+04	0.0E+00	1.9E+04	0.0E+00
Electricity	[kWh]	1.4E+04	0.0E+00	7.1E+03	0.0E+00	1.4E+04	0.0E+00	7.2E+03	0.0E+00

Table 5- Biochemical Factory (forecast for bacteria growth with glucose).

**BNaF 70 G**		**BNaF 60 G**		**BNaF 50 G**		**BKF 70 G**	
in	out	in	out	in	out	in	out
0.0E+00	9.1E+03	0.0E+00	9.1E+03	0.0E+00	8.9E+03	0.0E+00	9.1E+03
1.3E+04	1.1E+04	1.3E+04	1.1E+04	1.3E+04	1.1E+04	1.3E+04	1.1E+04
1.3E+04	4.1E+03	1.3E+04	4.1E+03	1.3E+04	4.1E+03	1.3E+04	4.1E+03
2.6E+04	2.6E+04	2.6E+04	2.6E+04	2.6E+04	2.6E+04	2.6E+04	2.6E+04
4.6E+03	2.0E+03	4.6E+03	2.0E+03	4.6E+03	2.0E+03	4.6E+03	2.0E+03
1.1E+05	1.1E+05	1.1E+05	1.1E+05	1.1E+05	1.1E+05	1.1E+05	1.1E+05
0.0E+00	0.0E+00	0.0E+00	0.0E+00	0.0E+00	0.0E+00	0.0E+00	0.0E+00
0.0E+00	0.0E+00	0.0E+00	0.0E+00	0.0E+00	0.0E+00	0.0E+00	0.0E+00
5.8E+03	4.3E+02	5.8E+03	4.3E+02	5.4E+03	2.2E+02	5.8E+03	4.3E+02
9.1E+03	1.1E+04	9.1E+03	1.1E+04	9.1E+03	1.1E+04	9.1E+03	1.1E+04
6.0E+02	2.3E+01	6.0E+02	2.3E+01	6.0E+02	2.3E+01	6.0E+02	2.3E+01
5.7E+01	2.3E+00	5.7E+01	2.3E+00	5.4E+03	2.2E+02	5.7E+01	2.3E+00
4.9E+00	3.9E+01	4.9E+00	3.9E+01	4.9E+00	3.9E+01	4.9E+00	3.9E+01
8.3E+00	2.5E+00	8.3E+00	2.5E+00	8.3E+00	2.5E+00	8.3E+00	2.5E+00
6.4E+02	3.3E+02	6.4E+02	3.3E+02	6.4E+02	3.3E+02	6.4E+02	3.3E+02
2.0E+07	0.0E+00	1.8E+07	0.0E+00	1.8E+07	0.0E+00	2.0E+07	0.0E+00
6.9E+05	0.0E+00	6.9E+05	0.0E+00	6.9E+05	0.0E+00	6.9E+05	0.0E+00
8.2E+04	0.0E+00	6.0E+04	0.0E+00	6.1E+04	0.0E+00	8.2E+04	0.0E+00
1.4E+04	0.0E+00	1.3E+04	0.0E+00	1.0E+08	0.0E+00	1.4E+04	0.0E+00
0.0E+00	2.2E+02	0.0E+00	2.2E+02	0.0E+00	2.2E+02	0.0E+00	2.2E+02
0.0E+00	4.0E+02	0.0E+00	4.0E+02	0.0E+00	4.0E+02	0.0E+00	4.0E+02
0.0E+00	1.1E+04	0.0E+00	1.1E+04	0.0E+00	1.1E+04	0.0E+00	1.1E+04
0.0E+00	4.1E+03	0.0E+00	4.1E+03	0.0E+00	4.1E+03	0.0E+00	4.1E+03
0.0E+00	1.1E+05	0.0E+00	1.1E+05	0.0E+00	2.2E+04	0.0E+00	1.1E+05
0.0E+00	4.0E+03	0.0E+00	4.0E+03	0.0E+00	2.0E+03	0.0E+00	4.0E+03
0.0E+00	2.6E+04	0.0E+00	2.6E+04	0.0E+00	2.6E+04	0.0E+00	2.6E+04
8.5E+04	0.0E+00	7.2E+04	0.0E+00	6.5E+04	0.0E+00	8.5E+04	0.0E+00
5.5E+03	0.0E+00	4.7E+03	0.0E+00	4.6E+03	0.0E+00	5.5E+03	0.0E+00
9.8E+00	0.0E+00	9.8E+00	0.0E+00	8.8E+00	8.8E+00	9.8E+00	0.0E+00

Table 6- Biochemical Factory (forecast for 100% CCU factory).

**Material balance and Energy demand**	**[Unit]**	**BNaF 70 CCU**	**BKF 70 CCU**	**BNaF 50 CCU**	**BNaF 60 CCU**

**Flow**		in	out	in	out	in	out	in	out
Formate	[kg/h]	0.0E+00	8.9E+03	0.0E+00	8.9E+03	0.0E+00	7.9E+03	0.0E+00	7.9E+03
CO	[kg/h]	1.1E+04	1.1E+04	1.1E+04	1.1E+04	1.1E+04	1.1E+04	1.1E+04	1.1E+04
CO_2	[kg/h]	1.1E+04	3.8E+03	1.1E+04	3.8E+03	1.1E+04	3.8E+03	1.1E+04	3.8E+03
O_2	[kg/h]	2.9E+05	2.9E+05	2.9E+05	2.9E+05	3.3E+05	3.3E+05	3.3E+05	3.3E+05
H_2 (BFG)	[kg/h]	2.0E+03	2.0E+03	2.0E+03	2.0E+03	2.0E+03	2.0E+03	2.0E+03	2.0E+03
Impurityg	[kg/h]	2.2E+04	2.2E+04	2.2E+04	2.2E+04	1.1E+06	1.1E+06	1.1E+06	1.1E+06
Glycerol	[kg/h]	0.0E+00	0.0E+00	0.0E+00	0.0E+00	0.0E+00	0.0E+00	0.0E+00	0.0E+00
Sodium/Potassiuum bicarbonate	[kg/h]	3.2E+03	6.4E+00	0.0E+00	0.0E+00	0.0E+00	0.0E+00	3.2E+03	6.4E+00
Sodium/Potassiuum hidroxide	[kg/h]	5.4E+03	1.8E+02	5.4E+03	1.8E+02	5.4E+03	7.8E+02	5.4E+03	7.8E+02
Water (process)	[kg/h]	9.1E+03	1.2E+04	9.1E+03	1.2E+04	9.1E+03	1.1E+04	9.1E+03	1.1E+04
Glucose	[kg/h]	0.0E+00	0.0E+00	0.0E+00	0.0E+00	0.0E+00	0.0E+00	0.0E+00	0.0E+00
Nitrogen (Bacteria gowth, NH_4OH)	[kg/h]	5.7E+01	3.4E+01	5.7E+01	3.4E+01	5.7E+01	3.1E+01	5.7E+01	3.1E+01
Amines (solvent, CCU)	[kg/h]	4.9E+00	3.9E+01	4.9E+00	3.9E+01	4.9E+00	3.9E+01	4.9E+00	3.9E+01
Salts (Bacteria gowth)	[kg/h]	8.3E+00	6.0E+00	8.3E+00	6.0E+00	8.3E+00	5.6E+00	8.3E+00	5.6E+00
H_2	[kg/h]	6.4E+02	3.3E+02	6.4E+02	3.3E+02	6.4E+02	3.3E+02	6.4E+02	3.3E+02
Polymer (PVA)	[kg/h]	1.9E+07	0.0E+00	1.9E+07	0.0E+00	1.6E+07	0.0E+00	1.9E+07	0.0E+00
Chilled Water	[kg/h]	7.0E+05	0.0E+00	7.0E+05	0.0E+00	7.0E+05	0.0E+00	7.0E+05	0.0E+00
Cooling Water	[kg/h]	7.7E+04	0.0E+00	7.7E+04	0.0E+00	4.3E+04	0.0E+00	7.1E+04	0.0E+00
Steam	[kg/h]	1.3E+04	0.0E+00	1.3E+04	0.0E+00	1.2E+04	0.0E+00	1.3E+04	0.0E+00
Hot Water	[kg/h]	0.0E+00	1.9E+02	0.0E+00	1.9E+02	0.0E+00	2.1E+02	0.0E+00	2.1E+02
Solid waste	[kg/h]	0.0E+00	6.1E+02	0.0E+00	6.1E+02	0.0E+00	7.5E+02	0.0E+00	6.2E+02
Aqueous waste	[kg/h]	0.0E+00	1.1E+04	0.0E+00	1.1E+04	0.0E+00	1.1E+04	0.0E+00	1.1E+04
CO (emisisons)	[kg/h]	0.0E+00	1.2E+02	0.0E+00	1.2E+02	0.0E+00	1.5E+02	0.0E+00	1.1E+02
CO_2 (emissions)	[kg/h]	0.0E+00	9.5E+05	0.0E+00	9.5E+05	0.0E+00	1.1E+06	0.0E+00	1.1E+06
Gaseous impurity (N_2)	[kg/h]	0.0E+00	5.0E+01	0.0E+00	5.0E+01	0.0E+00	5.0E+01	0.0E+00	5.0E+01
H_2 (emissions)	[kg/h]	0.0E+00	2.9E+05	0.0E+00	2.9E+05	0.0E+00	3.3E+05	0.0E+00	3.3E+05
O_2 (emissions)	[kg/h]	8.1E+04	0.0E+00	8.1E+04	0.0E+00	7.1E+04	0.0E+00	7.8E+04	0.0E+00
Utilities (total energy)	[kWh]	5.6E+03	0.0E+00	5.6E+03	0.0E+00	4.0E+03	0.0E+00	4.8E+03	0.0E+00
Electricity	[kWh]	9.8E+00	0.0E+00	9.8E+00	0.0E+00	9.8E+00	0.0E+00	9.8E+00	0.0E+00

1.3. Background process

The list of background processes was provided in Table 7.

Table 7- Background process of formate/formic acid production (Ecoinvent, cutoff).

Input/output	Unit	Region
formic acid production, methyl formate route	kg	RER
market for sodium formate	kg	RER
sodium formate production	kg	GLO
potash salt production	kg	RER
imidazole production	kg	RER Europe
electricity, low voltage, European attribute mix	kg	RER
market for tap water	kg	Europe without Switzerland
nutrient supply from ammonia, anhydrous, liquid	kg	RER
polyvinylchloride production, emulsion polymerisation	kg	RER
sodium chloride production, powder	kg	RER
treatment of municipal solid waste, incineration	kg	GB
treatment of biowaste by anaerobic digestion	kg	RoW
market for sodium hydroxide, without water, in 50% solution state	kg	GLO
potassium hydroxide production	kg	RER
sodium bicarbonate, to generic market for neutralising agent	kg	GLO
hydrogen production, steam reforming	kg	RER
glycerine production, from epichlorohydrin	kg	RER
tetrahydrofuran production	kg	RER
ethanolamine production	kg	RER

2. Techno-economic inventory

Since estimates of the capital expenditure (CapEx) were not available on SuperPro Designer® (v.13), then electrolyser costs were based on polymer electrolyte membrane (PEM) electrolyser for hydrogen production. According to IRENA forecasts, both PEM and anion exchange membranes (AEM) might cost less than USD 200/kW by 2050 [68,92]. The range of variability for the former systems was estimated in USD 306/kW to USD 4 748/kW [90,91]. Here, the minimum CapEx of USD 306/kW was used to estimate the potential cost of the electrolyser. Figure 1 showed the scheme of the former equipment.

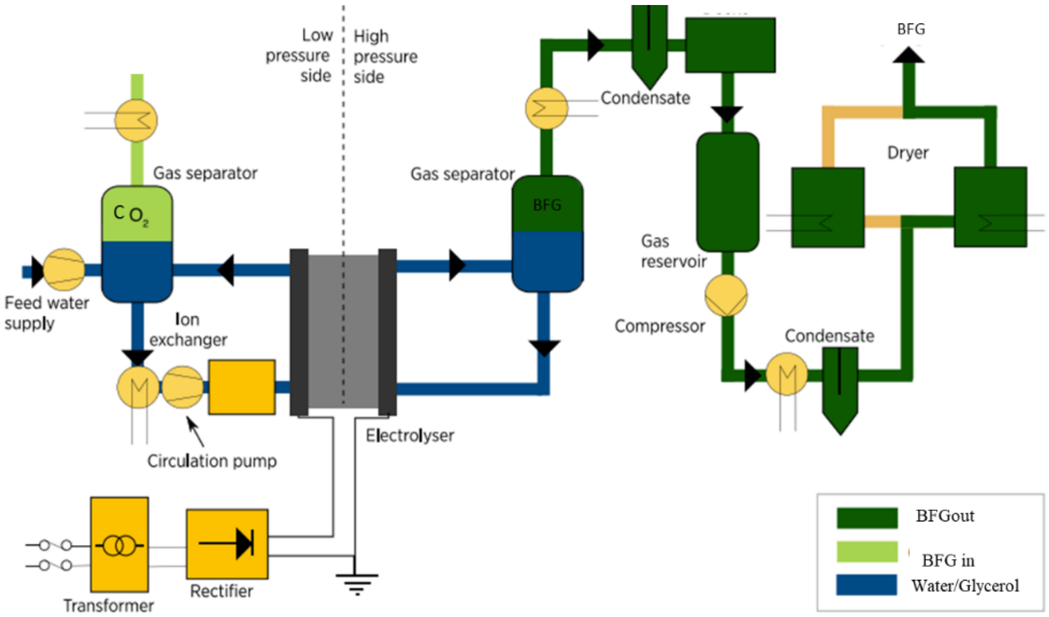

Figure 1- Potential industrial electrolyser.
Adapted from hydrogen production systems[68,92].

As discussed, the production of formate could lead to lower CapEx since some equipment could be avoided. However, this forecast is valuable to infer future costs of electrolysers.

The default values for TEA (SuperPro Designer ®) were summarized in Table 8.

Table 8- Summary of default values for TEA (SuperPro Designer ®).

**Year of analysis**	2024
**Currency**	USD
**Inflation rate**	4%
**Construction period**	30 months
**startup period**	4
**project lifetime**	30
**Intern rate of return**	7% (default)
**Inflation rate**	4%

Table 9 - Physical properties and costs for utilities.

Utility	Physical properties	Unit Cost		Reference
**Steam**	242 °C (high P),<br>152 °C (medium P)	0.0455	USD/ kW-h	[93]
	112 °C (low P)	0.0347	USD/ kW-h	[94]
**Hot Water***	80-43 °C; 1 bar	0.1000	USD/ kW-h	SuperPro Process Design software (v.13)
**Chilled Water**	5-25 °C; 1 bar	0.0295	USD/ kW-h	[95]
	5-10 °C; 1 bar	0.0295	USD/ kW-h	
**Cooling Water**	20-35 °C; 1 bar	0.0172	USD/ kW-h	[96]
	25-30 °C; 1 bar	0.00*	USD/ kW-h	
**Electricity**	low voltage	0.1000	USD/ kW-h	[37]

*Based on electricity cost.
**Energy recovery was considered for all scenarios.

Table 10 - Physical properties and costs for utilities.

Utility	Physical properties	Unit Cost		Reference
**Steam**	242 °C (high P),<br>152 °C (medium P)	0.0455	USD/ kW-h	[93]
	112 °C (low P)	0.0347	USD/ kW-h	[94]
**Hot Water***	80-43 °C; 1 bar	0.1000	USD/ kW-h	SuperPro Process Design software (v.13)
**Chilled Water**	5-25 °C; 1 bar	0.0295	USD/ kW-h	[95]
	5-10 °C; 1 bar	0.0295	USD/ kW-h	
**Cooling Water**	20-35 °C; 1 bar	0.0172	USD/ kW-h	[96]
	25-30 °C; 1 bar	0.00*	USD/ kW-h	
**Electricity**	low voltage	0.1000	USD/ kW-h	[37]

*Based on electricity cost.
**Energy recovery was considered for all scenarios.

Table 11- Prices per unit of auxiliary inputs/outputs.

Input/output cost	Price [$/ Unit]	Unit	Reference
Air	0	Nm^3	-
CO_2 -BFG	0	Nm^3	-
Gas impurities	0	Nm^3	-
H_2-BFG	0	Nm^3	-
H_2	0.10	Nm^3	[69,97]
$CaCl_2$	270.00	MT	[98]
Alginate	444.90	ton	[99]
Polyvinyl alcohol (PVA)-raw	2,120.00	ton	[100]
PVA -recycled	0	ton	-
Glycerol	784.66	ton	[101]
K_2CO_3	1351.00	ton	[102]
Na_2CO_3	278.80	ton	[103]
NH_4OH	154.22	ton	SuperPro
Glucose	453.59	ton	[104]
Batch Medium	30.00	ton	SuperPro
Amine solvents	1,280.00	ton	[105]
NaOH	444.90	MT	[106]
KOH	757.50	MT	[107]
RO Water	5.00	MT	[108]
Salts	1,360.78	ton	SuperPro
Water process	0.35	m^3(STP)	13
Demineralized Water	0.75		[108]
Wastes *			
Aqueous	2	ton	[109]
Solid	58.00(default)-140.00	ton	[110]
Gas (BFG) (+)	0 - 54	ton	[111–113]
Ash	10	ton	SuperPro
Formate (70%*wt*)	250	ton	[114]

(+) The production of chemical in this scenario was considered as a waste treatment, adding extra revenue to the factory. While other wastes were included on OpEx.

Table 11 - Weight factor for direct fixed capital (DFC) as function of the purchase cost of each equipment (PC) and direct cost (DC).

**Direct cost (DC)**		
Pipes, valves and fittings	0.19	xPC
Instrumentation	0.11	xPC
Insulation	0.13	xPC
Electrical facilities	0.07	xPC
Building	0.05	xPC
Yard improvements (structural supports and miscellaneous)	0.11	xPC
Auxiliary facilities (buildings)	0.05	xPC
Unlisted Equipment* (heat exchangers and tanks, process machinery)	0.47	xPC
**Indirect costs (IC)**		
Engineering and supervision	0.1	xDC
Construction labour	0.29	xDC
**Other costs (OC)**		
Contractor's fee	0.1	xDC
Contingency	0.1	xDC

Tax over the income was estimated in 25% .

Table 12 -Facility-dependent cost (FDC),
excluded from the first analysis and considered up to 10%xDFC for uncertainties.

Insurance	0	% xDFC
Local Taxes	0	% xDFC (minimum)
Factory expense	0	% xDFC

*Do not apply for the electrolysis since the cost of the equipment was estimated with additional facilities.
Depreciation (10y straight line).

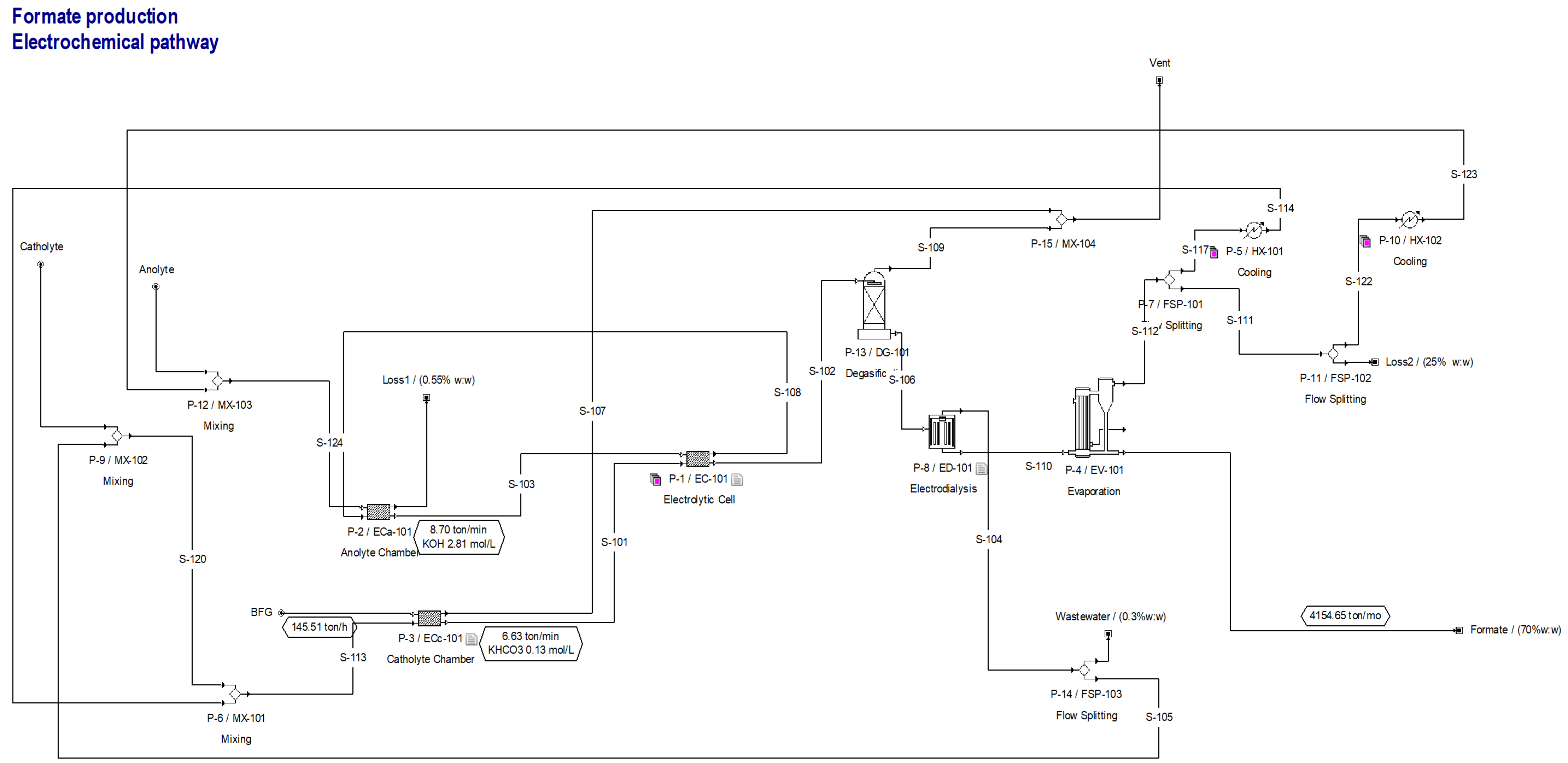
Formate production
Electrochemical pathway
Vent
S-123
S-114
Catholyte
Anolyte
S-109
P-15 / MX-104
S-117
P-5 / HX-101
Cooling
P-10 / HX-102
Cooling
S-122
P-7 / FSP-101
Splitting
S-112
S-111
P-13 / DG-101
Degasific
S-102
S-106
Loss2 / (25% w:w)
P-11 / FSP-102
Flow Splitting
Loss1 / (0.55% w:w)
P-12 / MX-103
Mixing
S-107
S-108
S-124
P-9 / MX-102
Mixing
S-103
P-1 / EC-101
Electrolytic Cell
P-8 / ED-101
Electrodialysis
S-110
P-4 / EV-101
Evaporation
P-2 / ECa-101
Anolyte Chamber
8.70 ton/min
KOH 2.81 mol/L
S-101
S-104
S-120
BFG
145.51 ton/h
P-3 / ECc-101
Catholyte Chamber
6.63 ton/min
KHCO3 0.13 mol/L
S-113
Wastewater / (0.3%w:w)
4154.65 ton/mo
Formate / (70%w:w)
P-6 / MX-101
Mixing
P-14 / FSP-103
Flow Splitting
S-105

a)

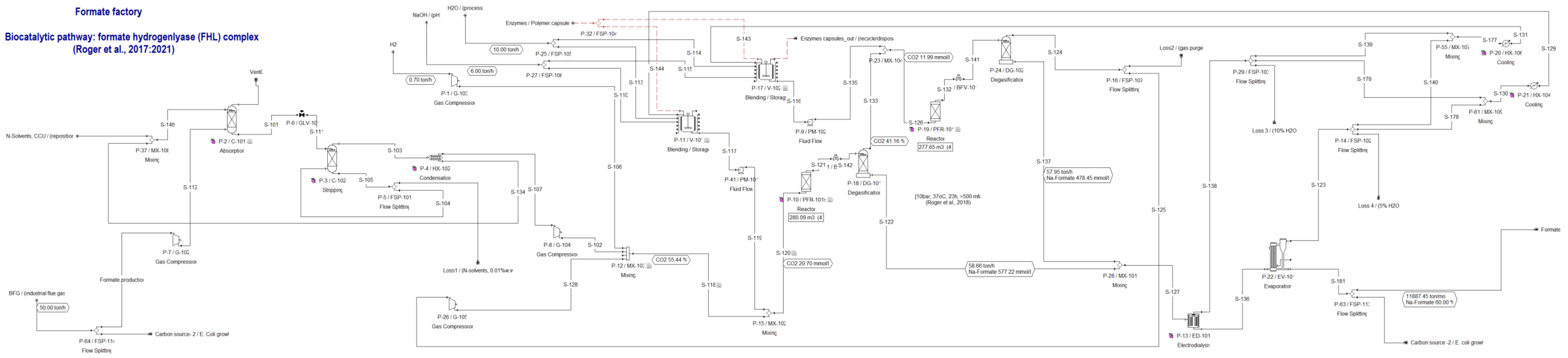
Formate factory
Biocatalytic pathway: formate hydrogenlyase (FHL) complex
(Roger et al., 2017:2021)

b)

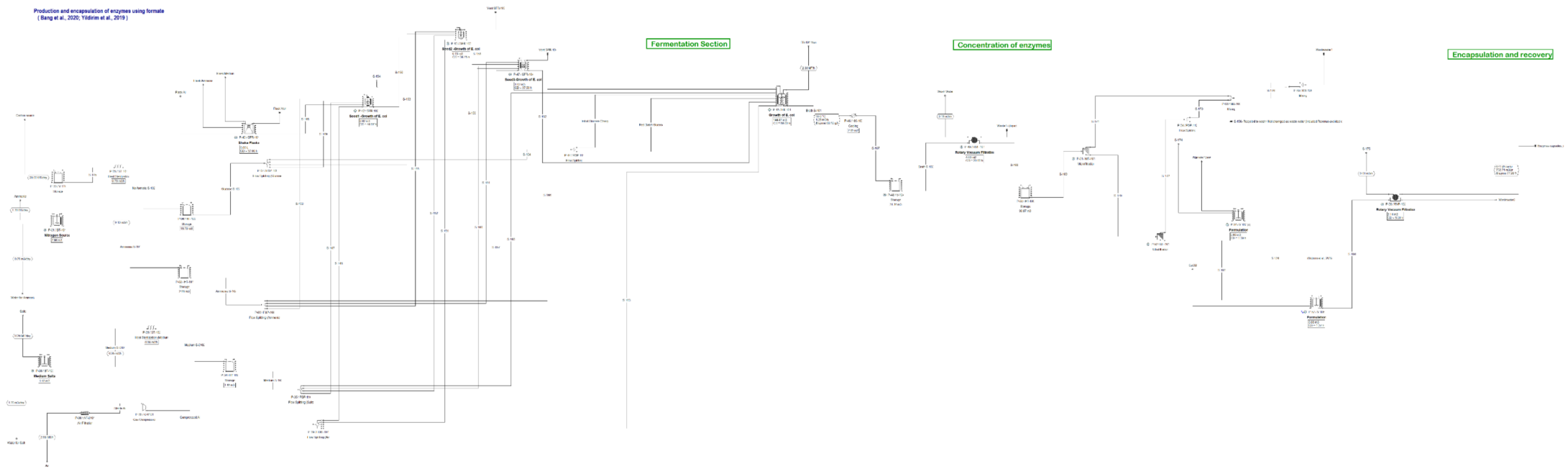

c)

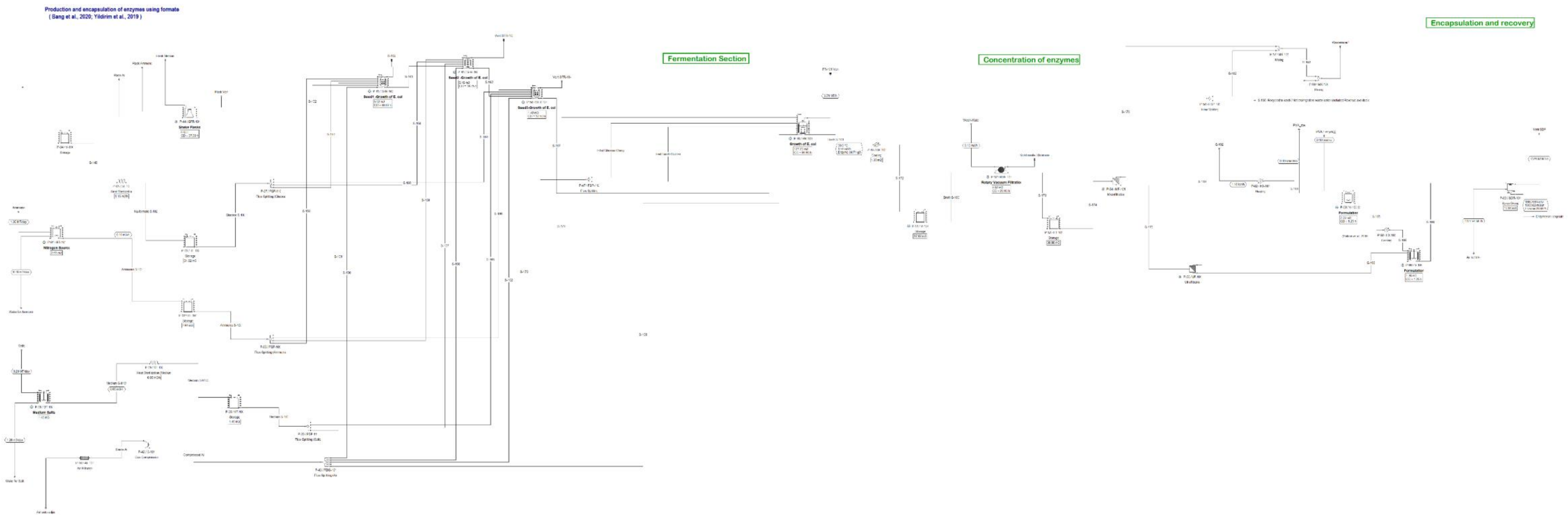

d)

Figure 2 --Process diagram of formate production. a) electrochemical pathway. B) Biochemical pathway. Enzymes production and encapsulation with alginate (c) and PVA(d)

3. Extra results

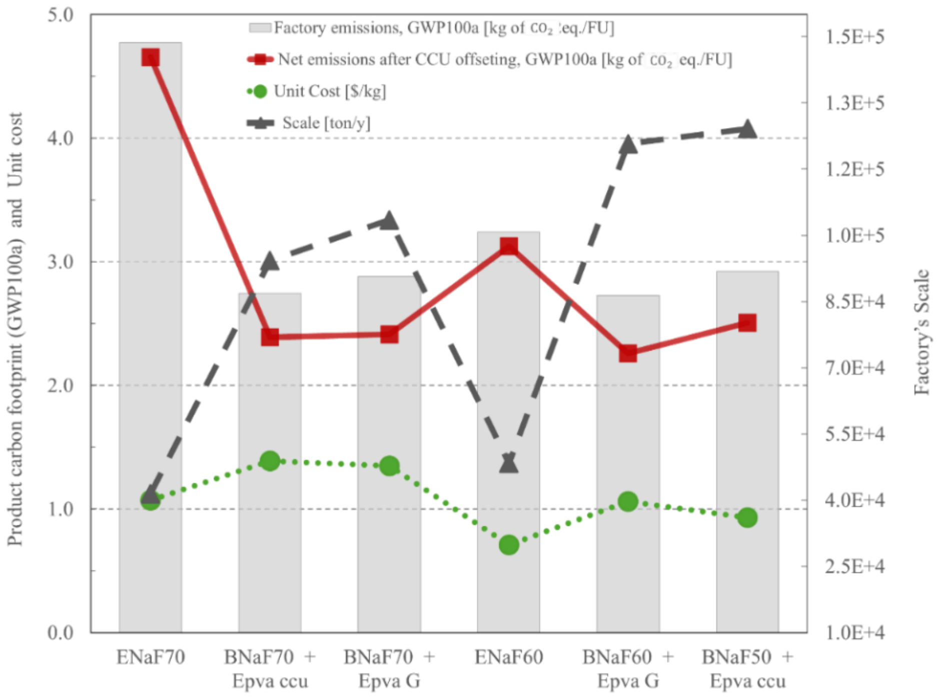

Figure 3- Summary of TEA and LCA for formate production.

4. Cost's uncertainty

From the perspective of buyer-supplier relationship, uncertainties in forecasting the product unit cost also may occur in CapEx and OpEx. The OpEx of biocatalyst production, for instance, was influenced by the carbon source used for bacterial growth and the demand of polymers that serve as physical supports for the enzymes. While both PVA and alginate beads can be recycled [39,88], the recovery of PVA has higher impact on the economic feasibility due to its cost, which can reach up to $2,160 (USA) per metric ton [100]. A scenario involving 50% recycled PVA (at zero cost) was potentially profitable, while the alginate scenario (with no recycling) was not, as indicated by the NPV of BNaF50% EPVA G and BNaF50%Ea, respectively.

Additionally, the CapEx of the biochemical system included extra costs associated with the production of the biocatalyst, which was attached to the CCU factory. The CO_2 capture system integrated into the biochemical factory also contributed to additional CapEx and OpEx (Fig. 2). If the biocatalyst could maintain its performance in the presence of inhibitory gases (e.g., CO, O_2), then the capture and recovery of CO_2 would not be necessary, reducing the CapEx by at least 39%—the total cost of compressing and capturing CO_2. However, to the best of our knowledge, the direct use of BFG to produce formate is limited; only pure gases have been tested in biochemical systems due to deactivation and inhibition issues caused by the presence of CO, O_2, and other gases [14]. Integrating CCU factories with high-grade CO_2-pipelines could present an opportunity, as absorption and stripping columns could be avoided.

The energy demand for producing formate was used to forecast the cost of a polymer electrolyte membrane electrolyser ($306/kW). This comprises of a set of equipment including: an electrolyser, 2 gas-separators, 1 gas drier, compressors, and heat exchangers (described in the Methodology section). Due to a lack of specific economic data, the model considered similarities between polymer electrolyte membrane (PEM) and anion exchange membrane (MEA) electrolysers to estimate the CapEx [90,91] of the industrial process. The estimation introduces uncertainties that could result in overestimating costs for the factory; therefore, a lower CapEx of the electrolyser system may be expected. Since additional costs related to the catalyst reposition were exclusively assigned to overheads, the TCI was lower for the ER than the BR. Additionally, the biocatalytic system required a fixed bed reactor (PBR), however the plug flow reactor was used to estimate the CapEx since PBRs are not available in SuperPro Designer ® - which could underestimate pumping requirements.

Raw materials, power demand, and revenues were also essential factors influencing the cash flow of the CCU factories. The BR required additional H_2 to produce formate, which contributed at least 7% to the OpEx of raw materials. The default purchase price of H_2 was

estimated at \$1.10/kg (= \$0.10/Nm^3), reflecting trends projected for 2040 [91]. Forecasts of industrial prices indicate that the highest and lowest purchase costs of H_2 since May 2023 were \$1.49/kg and \$0.68/kg [69]. This variability—approximately $\pm$50% —could significantly impact the profitability of the system.

Replacing KOH with NaOH resulted in higher profitability for both factories. In both the ER and BR systems, the purchase cost of raw inputs decreased from 698.53 USD/ton of KOH to 444.9 USD/ton of NaOH when producing NaF instead of KF. For ER, additional benefits might be observed in the OpEx if Na_2CO_3 is substituted with K_2CO_3. The purchase cost of carbonates changed from 1,348 USD/ton of K_2CO_3 [102] to 278.8 USD/ton of Na_2CO_3 [103]. The production of NaF instead of KF led to an outstanding reduction of raw materials costs by mitigating 34% of total OpEx (from 7.78E+07 to 5.14E+07 USD/y). Although the OpEx was reduced, the mitigation of those costs was not enough to achieve profitability in the system if the market grade (70% *wt* - NaF70%) of the final product was mandatory and excluded revenue gained from industrial flue gas treatment, indicated by + in each scenario. This suggests that potential uses of formate solutions at lower grades (60%*wt* and 50% *wt*) might support the economic feasibility of the CCU factory.

To reduce downstream separation energy costs and associated emissions, higher productivity might be required [115]. As, expected, energy savings from producing low-grade products resulted in economic and environmental benefits. In this context, exploring new applications for formate solutions below market grade could unveil opportunities for advancing formate production with CCUt.

Savings due to heat exchange were more critical for the BR than the ER. Although high demand of utilities was necessary to achieve the commercial grade of the final product in both systems, the biocatalytic factory required extra heat flows in the reactors to control the temperature of the biochemical processes at approximately 40°C. On the other hand, the

operational window for the electrolyser varied widely (1.36-7.92 kW/(kg/h) [23]), allowing for more precise forecasting of electricity demand, which could mitigate investment risk. The profitability of systems producing lower grade products (ENaF60%; BNaF50% $E_{PVA\ G}$) was achievable with a single revenue source.

Considering supply chain design, two markets emerge in the future: one for low-grade formate solutions and another for industrial flue gas treatment. Establishing a market for industrial flue gases (such as BFG) may be easier than adapting the market for low-grade product, which could require novel technologies for downstream applications. Additionally, reallocating fees currently paid for CO_2 emissions to charges for treating CO_2 in CCU factories could generate sufficient extra revenue, enabling the operation of a circular economy.

The location for launching CCU factories should be chosen with caution, considering both the availability of feedstock and raw auxiliary materials. The feedstock needs to be available for at least for 30 years to cover the usual lifetime of an industrial site. Energy costs are also critical for CCU factories to breakeven. Here, the cost of electricity was assumed to be 0.10 USD/kWh for both power and utilities. However, energy prices can vary significantly based on the region of investment. The comparison of different regions, using the price of UK's energy supply as reference, indicated that the highest savings in OpEx would be expected in China (<77%), followed by Brazil (<60%), USA (49%), and EU (43%)[36]. In EU countries, for instance, the range of energy cost can vary from 0.20 to 0.50 EUR/kWh [37] whereas in USA [38] and China[46], power costs for industrial processes can be reduced, being estimated between 0.07 to 0.10 USD/kWh from 2020 to 2023. Although the low price of energy in developing countries , as Brazil, can be promising, further investments in CCU policy might be necessary, mainly in infrastructure for supply chain designing. The overview of supply costs indicated that the USA and China may present better opportunities for CCU sites than EU region.

REFERENCES

(1) Malusare, D. U.; Ghumra, D. P.; Yadav, M. D. Bioconversion of CO_2 and Potential of Gas Fermentation for Mainstream Applications: Critical Advances and Engineering Challenges. Can J Chem Eng 2023, 101 (12), 6774–6791. https://doi.org/10.1002/cjce.24977.

(2) UNDESA. Sustainable Development Goals (SDGs); 2023. https://unosd.un.org/content/sustainable-development-goals-sdgs (accessed 2025-01-16).

(3) Bierbaumer, S.; Nattermann, M.; Schulz, L.; Zschoche, R.; Erb, T. J.; Winkler, C. K.; Tinzl, M.; Glueck, S. M. Enzymatic Conversion of CO_2 : From Natural to Artificial Utilization. Chem Rev 2023, 123 (9), 5702–5754. https://doi.org/10.1021/acs.chemrev.2c00581.

(4) Keep, M. ; J. I. ; W. M. Contribution of the Steel Industry to the UK Economy; UK, 2023. https://researchbriefings.files.parliament.uk/documents/CDP-2023-0016/CDP-2023-0016.pdf (accessed 2025-01-16).

(5) Business research insights. Sodium Formate Market Report; 2024. https://www.businessresearchinsights.com/market-reports/sodium-formate-market-102789 (accessed 2025-01-16).

(6) Yu, Z.; An, X.; Kurnia, I.; Yoshida, A.; Yang, Y.; Hao, X.; Abudula, A.; Fang, Y.; Guan, G. Full Spectrum Decomposition of Formic Acid over γ-Mo_2N-Based Catalysts: From Dehydration to Dehydrogenation. ACS Catal 2020, 10 (9). https://doi.org/10.1021/acscatal.0c00752.

(7) Sang, R.; Stein, C. A. M.; Schareina, T.; Hu, Y.; Léval, A.; Massa, J.; Turan, V.; Sponholz, P.; Wei, D.; Jackstell, R.; Junge, H.; Beller, M. Development of a Practical Formate/Bicarbonate Energy System. Nat Commun 2024, 15 (1), 7268. https://doi.org/10.1038/s41467-024-51658-2.

(8) Lee, J.; Kim, S. M.; Jeon, B. W.; Hwang, H. W.; Poloniataki, E. G.; Kang, J.; Lee, S.; Ra, H. W.; Na, J.; Na, J.-G.; Lee, J.; Kim, Y. H. Molar-Scale Formate Production via Enzymatic Hydration of Industrial off-Gases. Nature Chemical Engineering 2024, 1 (5), 354–364. https://doi.org/10.1038/s44286-024-00063-z.

(9) Kim, C.; Yoo, C. J.; Oh, H. S.; Min, B. K.; Lee, U. Review of Carbon Dioxide Utilization Technologies and Their Potential for Industrial Application. Journal of CO2 Utilization. 2022. https://doi.org/10.1016/j.jcou.2022.102239.

(10) Huang, Z.; Grim, R. G.; Schaidle, J. A.; Tao, L. The Economic Outlook for Converting CO2and Electrons to Molecules. Energy Environ Sci 2021, 14 (7). https://doi.org/10.1039/d0ee03525d.

(11) NREL. Economic Feasibility for CO_2 Utilization Data Visualization Tool; 2021. https://www.nrel.gov/bioenergy/co2-utilization-economics/ (accessed 2025-01-16).

(12) McDowall, J. S.; Murphy, B. J.; Haumann, M.; Palmer, T.; Armstrong, F. A.; Sargent, F. Bacterial Formate Hydrogenlyase Complex. Proceedings of the National Academy of Sciences 2014, 111 (38). https://doi.org/10.1073/pnas.1407927111.

(13) McDowall, J. S.; Hjersing, M. C.; Palmer, T.; Sargent, F. Dissection and Engineering of TheEscherichia Coli Formate Hydrogenlyase Complex. FEBS Lett 2015, 589 (20PartB), 3141–3147. https://doi.org/https://doi.org/10.1016/j.febslet.2015.08.043.

(14) Roger, M.; Reed, T. C. P.; Sargent, F. Harnessing Escherichia Coli for Bio-Based Production of Formate under Pressurized H 2 and CO 2 Gases. Appl Environ Microbiol 2021, 87 (21). https://doi.org/10.1128/AEM.00299-21.

(15) Yildirim, D.; Alagöz, D.; Toprak, A.; Tükel, S.; Fernandez-Lafuente, R. Tuning Dimeric Formate Dehydrogenases Reduction/Oxidation Activities by Immobilization. Process Biochemistry 2019, 85, 97–105. https://doi.org/10.1016/j.procbio.2019.07.001.

(16) Basso, A.; Serban, S. Industrial Applications of Immobilized Enzymes—A Review. Molecular Catalysis 2019, 479, 110607. https://doi.org/10.1016/j.mcat.2019.110607.

(17) Eş, I.; Vieira, J. D. G.; Amaral, A. C. Principles, Techniques, and Applications of Biocatalyst Immobilization for Industrial Application. Appl Microbiol Biotechnol 2015, 99 (5), 2065–2082. https://doi.org/10.1007/s00253-015-6390-y.

(18) Roger, M.; Brown, F.; Gabrielli, W.; Sargent, F. Efficient Hydrogen-Dependent Carbon Dioxide Reduction by Escherichia Coli. Current Biology 2018, 28 (1), 140-145.e2. https://doi.org/10.1016/j.cub.2017.11.050.

(19) Bang, J.; Hwang, C. H.; Ahn, J. H.; Lee, J. A.; Lee, S. Y. Escherichia Coli Is Engineered to Grow on CO2 and Formic Acid. Nat Microbiol 2020, 5 (12). https://doi.org/10.1038/s41564-020-00793-9.

(20) Bang, J.; Lee, S. Y. Assimilation of Formic Acid and CO2 by Engineered Escherichia Coli Equipped with Reconstructed One-Carbon Assimilation Pathways. Proc Natl Acad Sci U S A 2018, 115 (40). https://doi.org/10.1073/pnas.1810386115.

(21) Tao, Y.; Wang, H.; Wang, J.; Jiang, W.; Jiang, Y.; Xin, F.; Zhang, W.; Jiang, M. Strategies to Improve the Stress Resistance of Escherichia Coli in Industrial Biotechnology. Biofuels, Bioproducts and Biorefining. 2022. https://doi.org/10.1002/bbb.2358.

(22) Ewis, D.; Arsalan, M.; Khaled, M.; Pant, D.; Ba-Abbad, M. M.; Amhamed, A.; El-Naas, M. H. Electrochemical Reduction of CO2 into Formate/Formic Acid: A Review of Cell Design and Operation. Separation and Purification Technology. 2023. https://doi.org/10.1016/j.seppur.2023.123811.

(23) Van Den Bosch, B. ; F. M. C. ; S. K. J. P. ; B. M. ;Rawls, B. J. ; P. M. F. Electrochemical Production of Formate. WO 2021/152054 A1, 2020.

(24) Belsa, B.; Xia, L.; García de Arquer, F. P. CO2 Electrolysis Technologies: Bridging the Gap toward Scale-up and Commercialization. ACS Energy Lett 2024, 9 (9), 4293–4305. https://doi.org/10.1021/acsenergylett.4c00955.

(25) Paulillo, A.; Pucciarelli, M.; Grimaldi, F.; Lettieri, P. The Life-Cycle Environmental Performance of Producing Formate via Electrochemical Reduction of CO2in Ionic Liquid. Green Chemistry 2021, 23 (17). https://doi.org/10.1039/d1gc02306c.

(26) Bagemihl, I.; Cammann, L.; Pérez-Fortes, M.; van Steijn, V.; van Ommen, J. R. Techno-Economic Assessment of CO2 Electrolysis: How Interdependencies between Model Variables Propagate Across Different Modeling Scales. ACS Sustain Chem Eng 2023, 11 (27). https://doi.org/10.1021/acssuschemeng.3c02226.

(27) Pinto, A. S. S.; McDonald, L. J.; Galvan, J. L. H.; McManus, M. Improving Life Cycle Assessment for Carbon Capture and Circular Product Systems. International Journal of Life Cycle Assessment. 2024. https://doi.org/10.1007/s11367-023-02272-9.

(28) McDonald, L. H. G. J. E. C. S. S. P. A. M. N. I.-M. T. ; F. T. R. J. C. A. & ; M. M. Towards a Unified Carbon Accounting Landscape. hilosophical Transactions of the Royal Society A: Mathematical, Physical and Engineering Sciences 2024.

(29) Bhore, S. J. Paris Agreement on Climate Change: A Booster to Enable Sustainable Global Development and Beyond. Int J Environ Res Public Health 2016, 13 (11). https://doi.org/10.3390/ijerph13111134.

(30) McDowall, J. S.; Hjersing, M. C.; Palmer, T.; Sargent, F. Dissection and Engineering of the Escherichia Coli Formate Hydrogenlyase Complex. FEBS Lett 2015, 589 (20PartB), 3141–3147. https://doi.org/10.1016/j.febslet.2015.08.043.

(31) Towler, G. P.; Sinnott, R. K. Chemical Engineering Design : Principles, Practice and Economics of Plant and Process Design; 2007.

(32) Somoza-Tornos, A.; Guerra, O. J.; Crow, A. M.; Smith, W. A.; Hodge, B. M. Process Modeling, Techno-Economic Assessment, and Life Cycle Assessment of the Electrochemical Reduction of CO2: A Review. iScience. 2021. https://doi.org/10.1016/j.isci.2021.102813.

(33) Patel, G. H.; Havukainen, J.; Horttanainen, M.; Soukka, R.; Tuomaala, M. Climate Change Performance of Hydrogen Production Based on Life Cycle Assessment. Green Chemistry 2024, 26 (2). https://doi.org/10.1039/d3gc02410e.

(34) Incer-Valverde, J.; Korayem, A.; Tsatsaronis, G.; Morosuk, T. "Colors" of Hydrogen: Definitions and Carbon Intensity. Energy Conversion and Management. 2023. https://doi.org/10.1016/j.enconman.2023.117294.

(35) Sacchi, R.; Terlouw, T.; Siala, K.; Dirnaichner, A.; Bauer, C.; Cox, B.; Mutel, C.; Daioglou, V.; Luderer, G. PRospective EnvironMental Impact AsSEment (Premise): A Streamlined Approach to Producing Databases for Prospective Life Cycle Assessment Using Integrated Assessment Models. Renewable and Sustainable Energy Reviews 2022, 160. https://doi.org/10.1016/j.rser.2022.112311.

(36) CHEMANALYST. Sodium Formate Price Trend and Forecast; 2024. https://www.chemanalyst.com/Pricing-data/sodium-formate-1586 (accessed 2025-01-31).

(37) EC. EU Energy in Figures – Statistical Pocketbook 2023; Publications Office of the European Union, 2023. https://doi.org/doi/10.2833/502436.

(38) STATISTA. Average Retail Electricity Price for Industrial Consumers in the United States from 1970 to 2023; 2023. https://www.statista.com/statistics/190680/us-industrial-consumer-price-estimates-for-retail-electricity-since-1970/#:~:text=Industrial%20consumers%20of%20electricity%20in%20the%20United%20States,peaked%20at%208.3%20U.S.%20dollar%20cents%20per%20kilowatt-hour. (accessed 2024-05-22).

(39) Adamiak, K.; Sionkowska, A. State of Innovation in Alginate-Based Materials. Marine Drugs. 2023. https://doi.org/10.3390/md21060353.

(40) WEF. Net-Zero Challenge: The Supply Chain Opportunity; 2021. https://www3.weforum.org/docs/WEF_Net_Zero_Challenge_The_Supply_Chain_Opportunity_2021.pdf (accessed 2024-08-08).

(41) DESNT. Net Zero Innovation Portfolio and the Advanced Nuclear Fund.; UK, 2022. https://assets.publishing.service.gov.uk/media/646f13627dd6e70012a9b34c/nzip-anf-progress-report-2021-22.pdf (accessed 2024-06-26).

(42) Xu, D.; Li, K.; Jia, B.; Sun, W.; Zhang, W.; Liu, X.; Ma, T. Electrocatalytic CO2 Reduction towards Industrial Applications. Carbon Energy. 2023. https://doi.org/10.1002/cey2.230.

(43) Lees, E. W.; Bui, J. C.; Romiluyi, O.; Bell, A. T.; Weber, A. Z. Exploring CO2 Reduction and Crossover in Membrane Electrode Assemblies. Nature Chemical Engineering 2024, 1 (5), 340–353. https://doi.org/10.1038/s44286-024-00062-0.

(44) DESNZ. CCUS: non-pipeline transport and cross-border CO2 networks - call for evidence. https://www.gov.uk/government/calls-for-evidence/carbon-capture-usage-and-storage-ccus-non-pipeline-transport-and-cross-border-co2-networks/ccus-non-pipeline-transport-and-cross-border-co2-networks-call-for-evidence (accessed 2024-06-26).

(45) DESNZ. Factsheet: UK Carbon Border Adjustment Mechanism; 2023. https://www.gov.uk/government/consultations/addressing-carbon-leakage-risk-to-support-decarbonisation/outcome/factsheet-uk-carbon-border-adjustment-mechanism (accessed 2025-01-31).

(46) DESNZ; DBIS. The Carbon Capture and Storage Infrastructure Fund: an update on its design.

(47) WB. EMISSIONS TRADING IN PRACTICE: A Handbook on Design and Implementation, 2nd edition.; International Bank for Reconstruction and Development / The World Bank: Washington DC, 2021.

(48) ICAP. USA - California Cap-and-Trade Program. International carbon action partnership. https://icapcarbonaction.com/en/ets/usa-california-cap-and-trade-program (accessed 2025-01-16).

(49) EC. Evolution of the European carbon market. https://climate.ec.europa.eu/eu-action/eu-emissions-trading-system-eu-ets/development-eu-ets-2005-2020_en#evolution-of-the-european-carbon-market (accessed 2025-01-16).

(50) ISCC. ISCC Carbon Footprint Certification; 2024. https://www.iscc-system.org/wp-content/uploads/2024/03/DRAFT_ISCC-CFC_incl-CCSCCU_v1.1_March2024_0.1-1.pdf (accessed 2024-06-26).

(51) Pinto, A. S. S.; Elias, A. M.; Furlan, F. F.; Ribeiro, M. P. A.; Giordano, R. C.; Farinas, C. S. Strategies to Reduce the Negative Impact of Inhibitors in Biorefineries: A Combined Techno-Economic and Life Cycle Assessment. J Clean Prod 2022, 345. https://doi.org/10.1016/j.jclepro.2022.131020.

(52) European Commission. PEFCR Guidance Document - Guidance for Product Environmental Footprint Category Rules (PEFCRs); 2018.

(53) Shell. Catalysts Shell. https://catalysts.shell.com/en/Cansolv-co2-fact-sheet (accessed 2024-02-01).

(54) Li, L.; Maeder, M.; Burns, R.; Puxty, G.; Clifford, S.; Yu, H. The Henry Coefficient of CO2 in the MEA-CO2-H2O System. In Energy Procedia; 2017; Vol. 114. https://doi.org/10.1016/j.egypro.2017.03.1313.

(55) Cai, Y.; Wang, W.; Li, L.; Wang, Z.; Wang, S.; Ding, H.; Zhang, Z.; Sun, L.; Wang, W. Effective Capture of Carbon Dioxide Using Hydrated Sodium Carbonate Powders. Materials 2018, 11 (2). https://doi.org/10.3390/ma11020183.

(56) Ye, X.; Lu, Y. Kinetics of CO2 Absorption into Uncatalyzed Potassium Carbonate-Bicarbonate Solutions: Effects of CO2 Loading and Ionic Strength in the Solutions. Chem Eng Sci 2014, 116. https://doi.org/10.1016/j.ces.2014.05.050.

(57) Soini, J.; Ukkonen, K.; Neubauer, P. High Cell Density Media for Escherichia Coli Are Generally Designed for Aerobic Cultivations - Consequences for Large-Scale Bioprocesses and Shake Flask Cultures. Microb Cell Fact 2008, 7. https://doi.org/10.1186/1475-2859-7-26.

(58) Mori, M.; Marinari, E.; De Martino, A. A Yield-Cost Tradeoff Governs Escherichia Coli's Decision between Fermentation and Respiration in Carbon-Limited Growth. NPJ Syst Biol Appl 2019, 5 (1). https://doi.org/10.1038/s41540-019-0093-4.

(59) Sassenburg, M.; Kelly, M.; Subramanian, S.; Smith, W. A.; Burdyny, T. Zero-Gap Electrochemical CO2 Reduction Cells: Challenges and Operational Strategies for Prevention of Salt Precipitation. ACS Energy Lett 2023, 8 (1). https://doi.org/10.1021/acsenergylett.2c01885.

(60) Pittkowski, R.; Krtil, P.; Rossmeisl, J. Rationality in the New Oxygen Evolution Catalyst Development. Current Opinion in Electrochemistry. 2018. https://doi.org/10.1016/j.coelec.2018.11.014.

(61) Luo, G. S.; Pan, S.; Liu, J. G. Use of the Electrodialysis Process to Concentrate a Formic Acid Solution. Desalination 2002, 150 (3), 227–234. https://doi.org/10.1016/S0011-9164(02)00978-5.

(62) Jaimeferrer, J.; COUALLIER, E.; VIERS, P.; DURAND, G.; RAKIB, M. Three-Compartment Bipolar Membrane Electrodialysis for Splitting of Sodium Formate into

Formic Acid and Sodium Hydroxide: Role of Diffusion of Molecular Acid. J Memb Sci 2008, 325 (2), 528–536. https://doi.org/10.1016/j.memsci.2008.07.059.

(63) Mutel, C. Brightway: An Open Source Framework for Life Cycle Assessment. The Journal of Open Source Software 2017, 2 (12). https://doi.org/10.21105/joss.00236.

(64) TfS. The Product Carbon Footprint Guideline for Chemical Industry; 2024.

(65) Wernet, G.; Bauer, C.; Steubing, B.; Reinhard, J.; Moreno-Ruiz, E.; Weidema, B. The Ecoinvent Database Version 3 (Part I): Overview and Methodology. International Journal of Life Cycle Assessment 2016, 21 (9). https://doi.org/10.1007/s11367-016-1087-8.

(66) EC; Centre, J. R.; Andreasi Bassi, S.; Biganzoli, F.; Ferrara, N.; Amadei, A.; Valente, A.; Sala, S.; Ardente, F. Updated Characterisation and Normalisation Factors for the Environmental Footprint 3.1 Method; Publications Office of the European Union, 2023. https://doi.org/doi/10.2760/798894.

(67) Herman, J.; Usher, W. SALib: An Open-Source Python Library for Sensitivity Analysis. The Journal of Open Source Software 2017, 2 (9), 97. https://doi.org/10.21105/joss.00097.

(68) iea. Comparison of the emissions intensity of different hydrogen production routes, 2021. https://www.iea.org/data-and-statistics/charts/comparison-of-the-emissions-intensity-of-different-hydrogen-production-routes-2021 (accessed 2025-01-22).

(69) INTRATEC. Industrial Hydrogen Price | Industrial Utilities; 2024. https://www.intratec.us/products/water-utility-costs/commodity/industrial-hydrogen-price (accessed 2024-05-20).

(70) Yishai, O.; Goldbach, L.; Tenenboim, H.; Lindner, S. N.; Bar-Even, A. Engineered Assimilation of Exogenous and Endogenous Formate in Escherichia Coli. ACS Synth Biol 2017, 6 (9), 1722–1731. https://doi.org/10.1021/acssynbio.7b00086.

(71) Maghraby, Y. R.; El-Shabasy, R. M.; Ibrahim, A. H.; Azzazy, H. M. E.-S. Enzyme Immobilization Technologies and Industrial Applications. ACS Omega 2023, 8 (6), 5184–5196. https://doi.org/10.1021/acsomega.2c07560.

(72) Park, K.; Lee, K. R.; Ahn, S.; Park, H.; Moon, S.; Yoon, S.; Jung, K. D. Investigating the Catalytic Deactivation of a Pd Catalyst during the Continuous Hydrogenation of CO2 into Formate Using a Trickle-Bed Reactor. Catalysts 2024, 14 (3). https://doi.org/10.3390/catal14030187.

(73) Fernández-Caso, K.; Díaz-Sainz, G.; Alvarez-Guerra, M.; Irabien, A. Electroreduction of CO2: Advances in the Continuous Production of Formic Acid and Formate. ACS Energy Letters. 2023. https://doi.org/10.1021/acsenergylett.3c00489.

(74) Li, T.; Lees, E. W.; Zhang, Z.; Berlinguette, C. P. Conversion of Bicarbonate to Formate in an Electrochemical Flow Reactor. ACS Energy Lett 2020, 5 (8). https://doi.org/10.1021/acsenergylett.0c01291.

(75) Ramdin, M.; Morrison, A. R. T.; De Groen, M.; Van Haperen, R.; De Kler, R.; Irtem, E.; Laitinen, A. T.; Van Den Broeke, L. J. P.; Breugelmans, T.; Trusler, J. P. M.; Jong, W. De; Vlugt, T. J. H. High-Pressure Electrochemical Reduction of CO2 to Formic Acid/Formate: Effect of PH on the Downstream Separation Process and Economics. Ind Eng Chem Res 2019, 58 (51). https://doi.org/10.1021/acs.iecr.9b03970.

(76) Agency for Natural Resorces and Energy. Descriptions of Standard Heating Values and Carbon Emission Factor by Energy Sources (2018 Year Period) (Japanese); 2020.

(77) Conrado, R. J.; Gao, A. H. Integration of Fermentation and Gasification. US11097967B2, 2019. https://patents.google.com/patent/US11097967B2/en (accessed 2024-02-02).

(78) Hakka, L. E.; Ouimet, M. A. Method for Recovery of CO2 from Gas Streams. US7056482B2, 2003.

(79) Mohammadi, M.-R.; Larestani, A.; Schaffie, M.; Hemmati-Sarapardeh, A.; Ranjbar, M. Predictive Modeling of CO2 Solubility in Piperazine Aqueous Solutions Using Boosting Algorithms for Carbon Capture Goals. Sci Rep 2024, 14 (1), 22112. https://doi.org/10.1038/s41598-024-73070-y.

(80) Ooi, Z. L.; Tan, P. Y.; Tan, L. S.; Yeap, S. P. Amine-Based Solvent for CO2 Absorption and Its Impact on Carbon Steel Corrosion: A Perspective Review. Chinese Journal of Chemical Engineering. 2020. https://doi.org/10.1016/j.cjche.2020.02.029.

(81) Barker, G. Towers. The Engineer's Guide to Plant Layout and Piping Design for the Oil and Gas Industries 2018, 285–308. https://doi.org/10.1016/B978-0-12-814653-8.00011-4.

(82) Mumford, K. A.; Smith, K. H.; Anderson, C. J.; Shen, S.; Tao, W.; Suryaputradinata, Y. A.; Qader, A.; Hooper, B.; Innocenzi, R. A.; Kentish, S. E.; Stevens, G. W. Post-Combustion Capture of CO2: Results from the Solvent Absorption Capture Plant at Hazelwood Power Station Using Potassium Carbonate Solvent. In Energy and Fuels; 2012; Vol. 26. https://doi.org/10.1021/ef201192n.

(83) Furcas, F. E.; Pragot, W.; Chacartegui, R.; Afzal, W. Sodium Carbonate-Based Post Combustion Carbon Capture Utilising Trona as Main Sorbent Feed Stock. Energy Convers Manag 2020, 208. https://doi.org/10.1016/j.enconman.2020.112484.

(84) Knuutila, H.; Juliussen, O.; Svendsen, H. F. Kinetics of the Reaction of Carbon Dioxide with Aqueous Sodium and Potassium Carbonate Solutions. Chem Eng Sci 2010, 65 (23). https://doi.org/10.1016/j.ces.2010.07.018.

(85) Pinske, C.; Sargent, F. Exploring the Directionality of Escherichia Coli Formate Hydrogenlyase: A Membrane-bound Enzyme Capable of Fixing Carbon Dioxide to Organic Acid. Microbiologyopen 2016, 5 (5), 721–737. https://doi.org/10.1002/mbo3.365.

(86) Peters, K.; Sargent, F. Formate Hydrogenlyase, Formic Acid Translocation and Hydrogen Production: Dynamic Membrane Biology during Fermentation. Biochimica et Biophysica

Acta (BBA) - Bioenergetics 2023, 1864 (1), 148919. https://doi.org/10.1016/j.bbabio.2022.148919.

(87) Nguyen, H. K.; Minato, T.; Moniruzzaman, M.; Kiyasu, Y.; Ogo, S.; Yoon, K.-S. Selective Formate Production from H2 and CO2 Using Encapsulated Whole-Cells under Mild Reaction Conditions. J Biosci Bioeng 2023, 136 (3), 182–189. https://doi.org/10.1016/j.jbiosc.2023.06.002.

(88) Rahman, L.; Goswami, J. Poly(Vinyl Alcohol) as Sustainable and Eco-Friendly Packaging: A Review. J Packag Technol Res 2023, 7 (1). https://doi.org/10.1007/s41783-022-00146-3.

(89) Buchner, G. A.; Stepputat, K. J.; Zimmermann, A. W.; Schomäcker, R. Specifying Technology Readiness Levels for the Chemical Industry. Industrial and Engineering Chemistry Research. 2019. https://doi.org/10.1021/acs.iecr.8b05693.

(90) Saba, S. M.; Müller, M.; Robinius, M.; Stolten, D. The Investment Costs of Electrolysis – A Comparison of Cost Studies from the Past 30 Years. International Journal of Hydrogen Energy. 2018. https://doi.org/10.1016/j.ijhydene.2017.11.115.

(91) IRENA. GREEN HYDROGEN COST REDUCTION SCALING UP ELECTROLYSERS TO MEET THE 1.5°C CLIMATE GOAL H 2 O 2. 2020.

(92) IEA. Global Hydrogen Review 2024; 2024. https://iea.blob.core.windows.net/assets/89c1e382-dc59-46ca-aa47-9f7d41531ab5/GlobalHydrogenReview2024.pdf (accessed 2024-10-14).

(93) INTRATEC. Industrial Steam Cost | Industrial Utilities; 2024. https://www.intratec.us/products/water-utility-costs/commodity/industrial-steam-cost (accessed 2024-05-20).

(94) INTRATEC. Industrial Steam Cost (Low Pressure) | Industrial Utilities. 2024.

(95) INTRATEC. Chilled Water Cost | Industrial Utilities; 2024. https://www.intratec.us/products/water-utility-costs/commodity/chilled-water-cost (accessed 2024-05-20).

(96) INTRATEC. Cooling Water Cost | Industrial Utilities; 2024. https://www.intratec.us/products/water-utility-costs/commodity/cooling-water-cost (accessed 2024-05-20).

(97) WEICHENHAIN, U. ;KAUFMANN, M. ;HÖLSCHER, M. S. M. Going Global: An Update on Hydrogen Valleys and Their Role in the New Hydrogen Economy; EU, 2022. https://h2v.eu/analysis/statistics/financing/hydrogen-cost-and-sales-prices (accessed 2024-05-20).

(98) CHEMANALYST. Calcium Chloride Price Trend and Forecast; 2024. https://www.chemanalyst.com/Pricing-data/calcium-chloride-1297 (accessed 2024-05-20).

(99) PR. Sodium Alginate Price Trend and Forecast; 2023. https://www.procurementresource.com/resource-center/sodium-alginate-price-trends (accessed 2024-05-20).

(100) INTRATEC. Polyvinyl Alcohol Prices : Historical and Current; 2019. https://www.intratec.us/chemical-markets/polyvinyl-alcohol-price (accessed 2024-05-20).

(101) INTRATEC. Glycerol Prices | Historical and Current; 2024. https://www.intratec.us/chemical-markets/glycerol-price (accessed 2024-05-20).

(102) CHEMANALYST. Potassium Carbonate Price Trend and Forecast; 2024. https://www.chemanalyst.com/Pricing-data/potassium-carbonate-1164 (accessed 2024-05-20).

(103) INDEXBOX. Sodium Carbonate Market Analysis, Forecast Size, Trends and Insights.; 2024. https://www.indexbox.io/blog/sodium-carbonate-price-per-ton-in-august-2022/#:~:text=In%20August%202022%2C%20the%20sodium%20carbonate%20price%20per,US%29%2C%20rising%20by%202.3%25%20against%20the%20previous%20month. (accessed 2024-05-20).

(104) PR. Glucose Syrup Price Trend and Forecast; 2023. https://www.procurementresource.com/resource-center/glucose-syrup-price-trends (accessed 2024-05-20).

(105) INTRATEC. Monoethanolamine Prices | Historical and Current; 2024. https://www.intratec.us/chemical-markets/monoethanolamine-price (accessed 2024-05-20).

(106) PR. Sodium Hydroxide Price Trend and Forecast; 2022. https://www.procurementresource.com/resource-center/sodium-hydroxide-price-trends (accessed 2024-05-20).

(107) BA. Potassium Hydroxide Price Index; 2024. https://businessanalytiq.com/procurementanalytics/index/potassium-hydroxide-price-index/ (accessed 2024-05-20).

(108) INTRATEC. Demineralized Water Cost | Industrial Utilities; 2024. https://www.intratec.us/products/water-utility-costs/commodity/demineralized-water-cost (accessed 2024-05-20).

(109) Dore, M. H. Global Drinking Water Management and Conservation: Optimal Decision-Making; 2015. https://doi.org/10.1007/978-3-319-11032-5.

(110) STATISTA. Average Cost to Landfill Municipal Solid Waste in the United States ; USA, 2022. https://www.statista.com/statistics/692063/cost-to-landfill-municipal-solid-waste-by-us-region/ (accessed 2024-05-20).

(111) DESNZ. Carbon Prices for Use in Civil Penalties; UK Emissions Trading Scheme : UK, 2023. https://www.gov.uk/government/publications/determinations-of-the-uk-ets-carbon-price/uk-ets-carbon-prices-for-use-in-civil-penalties-2023 (accessed 2024-05-20).

(112) OCDE. Effective Carbon Rates 2023; OECD, 2023. https://doi.org/10.1787/b84d5b36-en.

(113) OCDE. Carbon Pricing in the United Kingdom; 2021. www.oecd.org/tax/tax-policy/carbon-pricing-background-notes.pdf (accessed 2024-05-20).

(114) MEDIUM. Sodium Formate Price Outlook | Q4 2022; 2022. https://medium.com/intratec-products-blog/sodium-formate-prices-latest-historical-data-in-several-countries-a5d68e48f4f9 (accessed 2024-05-20).

(115) Kibria Nabil, S.; McCoy, S.; Kibria, M. G. Comparative Life Cycle Assessment of Electrochemical Upgrading of CO2 to Fuels and Feedstocks. Green Chemistry 2021, 23 (2), 867–880. https://doi.org/10.1039/D0GC02831B.
